# Activated αβ T and reduced mucosa-associated invariant T cells in LGI1- and CASPR2-encephalitis

**DOI:** 10.1093/brain/awaf096

**Published:** 2025-03-17

**Authors:** Daniela Esser, Louisa Müller-Miny, Michael Heming, Manuela Paunovic, Martijn van Duijn, Ligia Abrante Cabrera, Katharina M Mair, Christine Strippel, Saskia Räuber, Justina Dargvainiene, Stjepana Kovac, Catharina C Gross, Nina Fransen, Robin van Steenhoven, Péter Körtvélyessy, Werner Stenzel, Romana Höftberger, Eric Bindels, Christian G Bien, Heinz Wiendl, Sven G Meuth, Jan Bauer, Nico Melzer, Maarten J Titulaer, Frank Leypoldt, Gerd Meyer zu Hörste, in cooperation with the EMC-AIE Study group, Juna M de Vries, Mariska M P Nagtzaam, Suzanne C Franken, Yvette S Crijnen, Juliette Brenner, Robin W van Steenhoven, Jeroen Kerstens, Marienke A A M de Bruijn, Anna E M Bastiaansen, Remco M Hoogenboezem, Sharon Veenbergen, Peter A E Sillevis Smitt

**Affiliations:** Institute of Clinical Chemistry, University Hospital Schleswig-Holstein Kiel/Lübeck, Kiel 24103, Schleswig-Holstein, Germany; Department of Neurology with Institute of Translational Neurology, University Hospital Münster, Münster 48143, North Rhine-Westphalia, Germany; Department of Neurology with Institute of Translational Neurology, University Hospital Münster, Münster 48143, North Rhine-Westphalia, Germany; Department of Neurology, Erasmus MC University Medical Center, Rotterdam 3000 DR, The Netherlands; Department of Neurology, Erasmus MC University Medical Center, Rotterdam 3000 DR, The Netherlands; Institute of Clinical Chemistry, University Hospital Schleswig-Holstein Kiel/Lübeck, Kiel 24103, Schleswig-Holstein, Germany; Department of Neuroimmunology, Center for Brain Research, Medical University of Vienna, Vienna 1090, Austria; Department of Neurology with Institute of Translational Neurology, University Hospital Münster, Münster 48143, North Rhine-Westphalia, Germany; Department of Neurology with Institute of Translational Neurology, University Hospital Münster, Münster 48143, North Rhine-Westphalia, Germany; Department of Neurology, Medical Faculty and University Hospital Düsseldorf, Heinrich Heine University Düsseldorf, Düsseldorf 40225, North Rhine-Westphalia, Germany; Institute of Clinical Chemistry, University Hospital Schleswig-Holstein Kiel/Lübeck, Kiel 24103, Schleswig-Holstein, Germany; Department of Neurology with Institute of Translational Neurology, University Hospital Münster, Münster 48143, North Rhine-Westphalia, Germany; Department of Neurology with Institute of Translational Neurology, University Hospital Münster, Münster 48143, North Rhine-Westphalia, Germany; Department of Pathology, Amsterdam UMC, Amsterdam 1098XH, The Netherlands; Department of Pathology, UMC Utrecht, Utrecht 2521CB, The Netherlands; Department of Neurology, Erasmus MC University Medical Center, Rotterdam 3000 DR, The Netherlands; Department of Neurology, Center for ALS and other Motor Neuron Disorders, Charité—Universitätsmedizin Berlin, Corporate Member of Freie Universität Berlin, Humboldt-Universität zu Berlin, and Berlin Institute of Health, Berlin 10117, Germany; Department of Neuropathology, Charité—Universitätsmedizin Berlin, corporate member of Freie Universität Berlin and Humboldt-Universität zu Berlin, Berlin 10117, Germany; Department of Neuroimmunology, Center for Brain Research, Medical University of Vienna, Vienna 1090, Austria; Department of Hematology, Erasmus MC University Medical Center, Rotterdam 3000 DR, The Netherlands; Department of Epileptology, Krankenhaus Mara, Bethel Epilepsy Center, Medical School OWL, Bielefeld University, Bielefeld 33501, North Rhine-Westphalia, Germany; Department of Neurology and Neurophysiology, Medical Center—University of Freiburg, Freiburg 79104, Baden-Wuerttemberg, Germany; Department of Neurology with Institute of Translational Neurology, University Hospital Münster, Münster 48143, North Rhine-Westphalia, Germany; Department of Neurology, Medical Faculty and University Hospital Düsseldorf, Heinrich Heine University Düsseldorf, Düsseldorf 40225, North Rhine-Westphalia, Germany; Department of Neuroimmunology, Center for Brain Research, Medical University of Vienna, Vienna 1090, Austria; Department of Neurology with Institute of Translational Neurology, University Hospital Münster, Münster 48143, North Rhine-Westphalia, Germany; Department of Neurology, Medical Faculty and University Hospital Düsseldorf, Heinrich Heine University Düsseldorf, Düsseldorf 40225, North Rhine-Westphalia, Germany; Department of Neurology, Erasmus MC University Medical Center, Rotterdam 3000 DR, The Netherlands; Institute of Clinical Chemistry, University Hospital Schleswig-Holstein Kiel/Lübeck, Kiel 24103, Schleswig-Holstein, Germany; Department of Neurology, University Hospital Schleswig-Holstein, Kiel 24103, Schleswig-Holstein, Germany; Department of Neurology with Institute of Translational Neurology, University Hospital Münster, Münster 48143, North Rhine-Westphalia, Germany

**Keywords:** autoimmune encephalitis, leucin-rich glioma inactivated 1, contactin-associated protein 2, single-cell transcriptomics, mucosa-associated invariant T cells, flow cytometry

## Abstract

Anti-leucine-rich glioma inactivated-1 (LGI1) and anti-contactin-associated-protein-2 (CASPR2) autoimmune encephalitis (AIE) are common and characterized by pathogenic antibodies targeting neuronal autoantigens. However, the drivers of the antibody-secreting cells and involvement of T cells remain unresolved. We performed single-cell RNA sequencing of fresh CSF and parallel blood samples of 15 patients with LGI1-AIE (*n* = 9) and CASPR2-AIE (*n* = 6) compared with control patients [multiple sclerosis (*n* = 15) and idiopathic intracranial hypertension (*n* = 18)]. We validated our observations in independent cohorts using flow cytometry of CSF and blood. We confirmed autoantibody specificity using recombinant human monoclonal antibodies.

In comparison to idiopathic intracranial hypertension and multiple sclerosis controls, we observed clonal CSF-specific antibody-secreting cell expansion in LGI1/CASPR2-AIE despite mostly normal CSF findings. Antibody-secreting cells were dominantly plasmablasts and transcribed IgG4 and IgG1/2 heavy chains. Expanded clones showed signs of affinity maturation and bound the respective neuronal autoantigen. Within CD4 and CD8 T-cell clusters, CD4 and CD8 central memory T cells were activated, clonally restricted and expanded. T-cell clones were often shared between CSF and blood. We also observed a shift of natural killer cells and loss of mucosa-associated invariant T (MAIT) cells in the CSF of LGI1-AIE and the blood of LGI1- and CASPR2-AIE compared with idiopathic intracranial hypertension and multiple sclerosis controls. MAIT-like T cells were detected in autopsied brains of LGI1- and CASPR2-AIE patients, and mice lacking MAIT cells displayed an increased antibody seroconversion and higher titres following active LGI1/CASPR2 immunization.

Our data: (i) confirm the intrathecal antigen-specific plasma cell expansion in LGI1- and CASPR2-AIE in a large cohort of untreated AIE patients; (ii) suggest that activated and expanded central memory CD4 and CD8 T cells in the CSF participate in disease pathogenesis; and (iii) implicate invariant T-cell receptor-expressing lymphocytes in the brain, CSF and blood in disease pathogenesis.

## Teaser

Esser *et al.* investigate the mechanisms underlying two rare subtypes of autoimmune encephalitis associated with seizures and neurocognitive deficits, identifying immune cell changes including B cell clonal expansion and downregulation of MAIT cells.

## Introduction

Anti-leucine-rich glioma inactivated 1 (LGI1) encephalitis and contactin-associated protein 2 (CASPR2) autoimmunity are two autoantibody-defined subgroups of autoimmune encephalitis (AIE)^1^ often manifesting as limbic encephalitis in elderly patients.^1,2^ Their clinical phenotypes are distinct, and evidence from direct antibody infusion murine models suggests that these clinical differences are caused by autoantigen-specific modes of antibody-mediated synaptic dysfunction.^3-6^ However, the mechanisms, propagation and trans-compartmental cross-talk of the underlying cellular autoimmune reaction in patients are considerably less well understood. Both diseases are remarkable for their lack of overt inflammatory changes in the CSF.^7,8^ Only 11% of patients have mild lymphocytic pleocytosis, and <25% of patients show CSF restricted oligoclonal bands^9^ with similar results in CASPR2 autoimmunity.^10^ In addition to the presence of antibody-secreting B cells^8,11^ in the CSF, both diseases share immunological features strongly hinting at involvement of T lymphocytes in pathogenesis: (i) a strong human leukocyte antigen (HLA) class II association^12-15^; and (ii) autoantibodies are mostly of the IgG4 isotype,^16,17^ and the class-switch to IgG4 autoantibodies requires CD4^+^ T-cell help.^18^ In summary, there is evidence for a role of intrathecal, compartmentalized antigen-specific T- and B-cell immune reactions in these diseases.

Therefore, we aimed at better characterizing the intrathecal and systemic immune compartment in LGI1- and CASPR2-AIE in comparison to idiopathic intracranial hypertension (IIH) and multiple sclerosis (MS) disease control patients. We performed a cross-compartment immune cell characterization of a representative, large, prospectively recruited, immunologically untreated, multicentre cohort of patients with LGI1- or CASPR2-AIE. We integrated single-cell RNA sequencing (scRNA-seq) with flow cytometry of CSF and blood samples in independent cohorts, analysis of autopsy brains and functional studies (focused on the role of invariant T-cell receptor lymphocytes) in a murine immunization model of CASPR2- and LGI1-encephalitis.

## Materials and methods

More detailed methods are provided in the Supplementary material.

### Prospective patient recruitment, CSF collection and retrospective data collection: cohort 1

All patients with suspected AIE or antibody-confirmed LGI1-/CASPR2-AIE at the centres in Münster, Rotterdam and Kiel were prospectively screened for inclusion into the study. Formal inclusion criteria for AIE patients were as follows: (i) fulfilment of the Gauss criteria^1^ for probable AIE extending the time frame for time since onset from 3 to 4 months; (ii) detection of LGI1-/CASPR2 antibodies in serum and/or CSF with suitable cell-based and tissue-based assays, as previously described^19,20^; (iii) receiving lumbar puncture for diagnostic purposes; (iv) immunotherapy naive except for a maximum of 1 day of steroid treatment; and (v) written informed consent to participate. All patients were of Caucasian ethnicity. The study was performed in accordance with the Declaration of Helsinki and was approved by: (i) the ‘Ethikkommission der Ärztekammer Westfalen-Lippe (ÄKWL) und der Westfälischen-Wilhelms-Universität’ (Ethics Committee of the Board of Physicians of the Region Westfalen-Lippe and of the Westfälische Wilhelms-University Münster), under reference number 2015-522-f-S, in Münster; (ii) the ‘Erasmus MC Medical Ethical Research Board’, under reference numbers MEC-2015-397, MEC-2020-0418 and MEC-2020-0650, in Rotterdam; and (iii) the ‘Ethikkommission’ of the medical faculty Kiel, reference numbers D498/19, B337/13, B300/19 and D578/18. The IIH patient cohort had been reported, in part, previously^21^ or were recruited for the present study and also fulfilled the inclusion criteria iii–v.

For the flow cytometry confirmation cohort 2, we retrospectively screened all patients who were admitted to the University Hospital Münster between 2011 and 2020 and received a diagnostic lumbar puncture, including flow cytometry, for the ICD-10 diagnoses G04.*, G13.1 or G93.2. Exclusion criteria, sample handling and details on retrospective sampling in cohorts 2 and 3 are provided in the Supplementary material.

### Single-cell RNA sequencing

Single-cell suspensions were loaded onto the Chromium Single Cell Controller using the Chromium Single Cell GEM, Library & Gel Bead Kits (10× Genomics) with chemistry version V1 and V1.1 for 5′ reagents and with V3 and V3.1 for 3′ reagents. Sample processing and library preparation were performed according to the manufacturer’s instructions, and sequencing was carried out on an Illumina Nextseq 500 or an Illumina Novaseq 6000 with 2 × 150 or 2 × 100 read set-up.

### Single-cell RNA sequencing bioinformatic analyses

The preprocessing of scRNA data was performed with the 10× Genomics’ Cell Ranger software v.8.0.1 using the reference GRCh38 v.2024-A. The resulting filtered feature-barcode matrix files were analysed with the R package Seurat v.5.1.0.^22^ To minimize the number of doublets, empty cells and cells with a transcriptome in low quality, only cells harbouring between 300 and 2500 RNA features and <5% mitochondrial RNA were selected for further processing. All genes with a detected expression in <0.1% of the cells and mitochondrial and ribosomal genes (comprising all genes having names starting with MT-, RPS, RPL or RNA\\d8S5) were not considered for further analyses. Afterwards, data were log-normalized, variable features identified, and features scaled and centred in the dataset. After performing a principal component analysis dimensionality reduction with the RunPCA function, the expression values were corrected for effects caused by different library preparation kits (3′ versus 5′) and different sampling locations using the R package Harmony v.1.2.0.^23^ In the final steps, the uniform manifold approximation and projection (UMAP) dimensional reduction was performed with the RunUMAP function, a shared nearest neighbour graph was created with the FindNeighbors function, and clusters were identified with a resolution of 0.1 using the FindClusters function. The clusters were annotated with the R function SingleR^24^ v.2.6.0 on the Human Primary Cell Atlas reference dataset, followed by a manual correction using marker genes computed with the R function FindMarkers and the percentage of each cell type predicted with the tool Azimuth v.0.5.0 using the human peripheral blood mononuclear cell (PBMC) reference.^22^ B- and T-cell clusters were reclustered with a resolution of 0.2 and 0.5, respectively. The annotation of the B and T cells was performed with the same strategy as for the main clustering except that Azimuth with a tonsil reference^25^ was used for the B cells. The same method was applied for reclustering and annotation of the T-cell cluster with gamma delta T cells (gdT), natural killer cells (NK), mucosal-associated invariant T cell (MAIT) and innate lymphoid cells.

### Cloning antibodies and verification of antigen specificity

Corresponding full-length consensus sequences of variable heavy (VH) and light (VL) chains including signal peptide sequences of the top five to nine most abundant, clonally expanded B cells in the CSF sample of five randomly selected patients (three LGI1- and two CASPR2-AIE) were determined from 5′ VDJ (variable–diversity–joining) 10× single-cell information. Cloning strategy, vectors and expression/purification strategies were kindly provided by M. Peipp, Kiel and done as described previously.^26^ Antigen specificity was confirmed using cell-based assays as previously reported.^19,20^ Human serum (1:40) from AIE patients or healthy subjects was used as positive or negative controls, respectively.

### Flow cytometry

Flow cytometry raw data from all treatment-naive patients from cohort 2 were analysed. All CSF cell samples collected during regular working hours at the centre in Münster were analysed routinely and promptly by flow cytometry using a Navios flow cytometer (Beckman Coulter) and an antibody panel described previously.^27^ Briefly, blood cells were lysed using VersaLyse buffer, and CSF cells were stained using the following anti-human antibodies (BioLegend; clone names indicated): CD3 (UCHT1); CD4 (13B8.2); CD8 (B9.11); CD14 (RMO52); CD16 (3G8); CD19 (J3-119); CD25 (B1.49.9); CD27 (1A4CD27); CD45 (J.33); CD45RA (ALB11); CD56 (N901, NCAM16.2); CD127 (R34.34); CD138 (B-A38); and HLA-DR (Immu-357). Cell population size was defined as the number of gated cell events relative to the events of the corresponding parent gate.

### Human CNS multiplex immunofluorescence labelling

Histological paraffin sections of brain and meningeal tissue from autopsied patients with the diagnosis CASPR2-AIE (*n* = 3) and LGI1-AIE (*n* = 3) were included. Sections were localized in the uncal and hippocampal region. Immunofluorescence labelling of sections was performed using markers for CD3 (Neomarkers, #RM9107-S), CD8 (Dako M7103), CD4 (Cell Signaling, #48274), TCR δ (SantaCruz, #sc-100289) and CD161 (Abcam, #ab302564). The staining procedure was executed in accordance with the Akoya Fluorescent Multiplex kit protocol.^28,29^ To quantify, cells were scanned with the Vectra Polaris Automated Quantitative Pathology Imaging system from Perkin Elmer and quantified with Qupath software. To this end, cells were detected by nuclear staining (DAPI) (Supplementary material).

### Mouse immunization and evaluation

All animal experiments were approved by the local authorities (Landesamt für Natur, Umwelt und Verbraucherschutz Nordrhein-Westfalen; Approval ID: 84-02.04.2022.A336). Every effort was made to minimize the number of animals used and to avoid stress and suffering of the animals by strictly following the ARRIVE guidelines. The mice were housed in groups, had a 12 h–12 h light–dark cycle, and food and water were available *ad libidum*. Homozygous MR1-deficient mice (MR1 AIE; LGI1 *n* = 3, CASPR2 *n* = 5) and C57BL/6 mice (C57BL6 AIE; LGI1 *n* = 2; CASPR2 *n* = 5) (10–15 weeks old, male and female) were immunized twice with recombinant mouse CNTNAP2 (>95% purity) or LGI1 protein (>90% purity) (Cntnap2-3316M, LGI1-9069M, Creative Biomart) emulsified in Complete Freund’s Adjuvant and supplemented with *Mycobacterium tuberculosis* H37Ra (4 mg/ml). Mice were immunized subcutaneously on the back with 100 μg of the protein in the emulsion mixture. Mice in the control groups, including homozygous MR1-deficient (MR1 control; *n* = 5) and C57BL/6 (C57BL6 control; *n* = 5) mice, received an emulsion mixture of Complete Freund’s Adjuvant and the same volume of PBS. All mice were injected intraperitoneally with 250 ng of pertussis toxin (Sigma) on the day of immunization and 48 h later. Serum from all mice was collected on the 28th day to determine antibody titres. A subset of mice (MR1 AIE, *n* = 6; C57BL6 AIE, *n* = 4; MR1 control, *n* = 5; C57BL6 control, *n* = 5) were evaluated 3–1 days prior, at 12–14 days and at 25–28 days after immunization and underwent behavioural tests to evaluate locomotor activity, anxiety levels and memory. All tests were evaluated by an automated program (Noldus Ethovision^30^) with manual correction. The Open Field test assessed locomotor activity and exploratory behaviour of mice.^31^ Animals were tested in the open field arena, and the distance travelled, velocity, and time spent in the centre were measured. Further detail on behavioural testing and murine CNS flow cytometry is provided in the Supplementary material.

## Results

### Sample acquisition, and patient and CSF characteristics of LGI1-AIE and CASPR2-AIE patients

**Figure 1:**
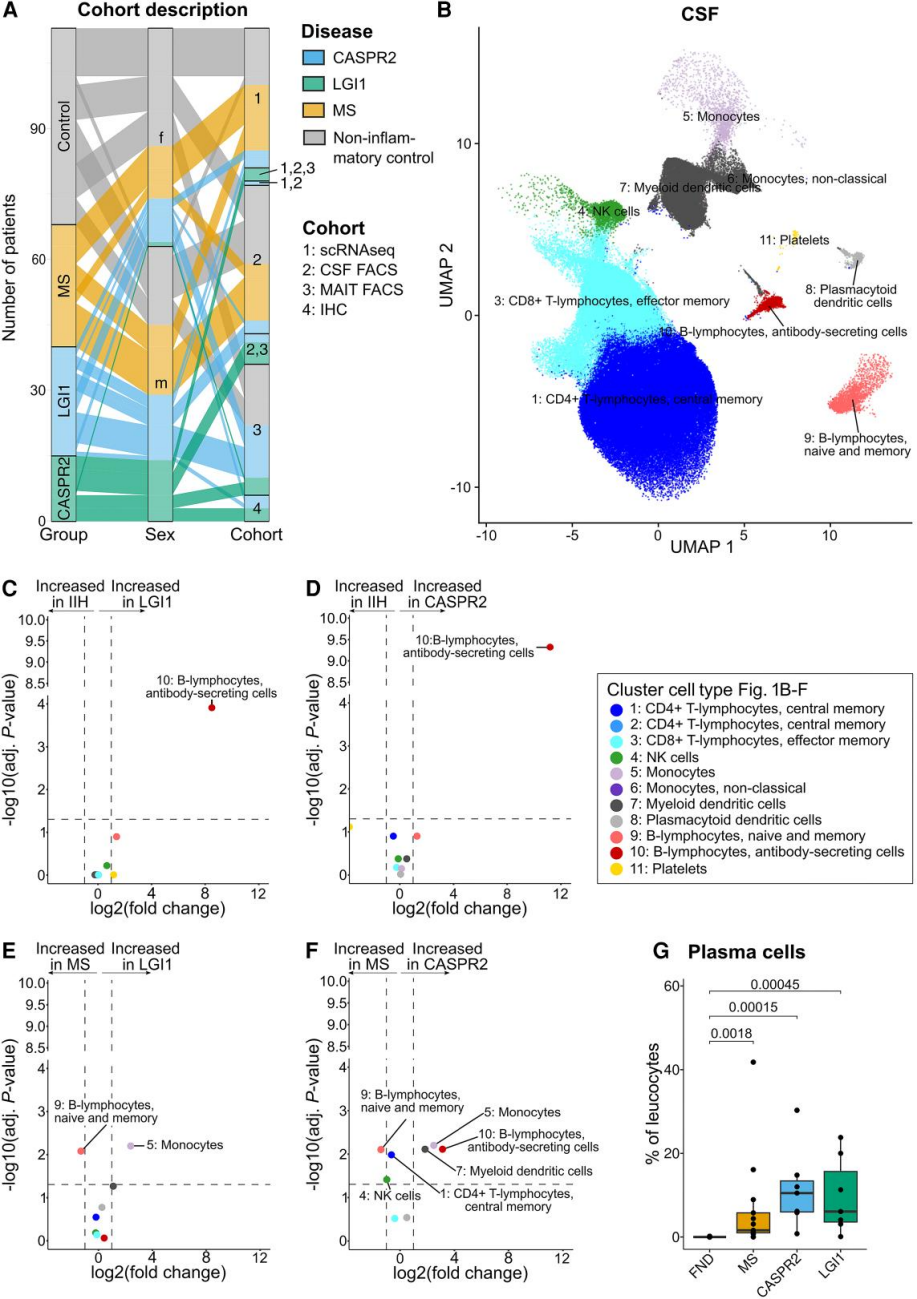
**Single-cell transcriptomics and flow cytometry identified expansion of plasma cells as a hallmark of LGI1-/CASPR2-AIE.** (**A**) Sankey diagram showing overlap between LGI1-AIE, CASPR2-AIE, non-inflammatory-disease controls (IIH in cohort 1, functional disorder in cohort 2 and healthy controls in cohort 3) across the four sample cohorts. IHC = immunohistochemistry analysis of formalin-fixed, paraffin-embedded (FFPE) autopsy brain tissue. (**B**) Uniform manifold approximation and projection (UMAP) plot depicting the cell-type clusters of CSF cells. The second cell cluster included only PBMCs and is therefore shown only in Supplementary Fig. 1D. (**C**–**F**) Comparison of the relative cell-type abundance between LGI1 and IIH (**C**), CASPR2 and IIH (**D**), LGI1 and MS (**E**) and CASPR2 and MS (**F**). (**G**) Flow cytometry validation: relative percentage of plasma cells (%CD3^−^CD19^+^CD138^+^) quantified as percentages of all lymphocytes in CSF cells of the second cohort. Statistical significance was determined by the Kruskal–Wallis test with Dunn’s *post hoc* test and adjusted with the Benjamini–Hochberg method. FACS = fluorescence-activated cell sorting; FND = functional neurological disorders; IIH = idiopathic intracranial hypertension; MAIT = mucosal-associated invariant T cell; MS = multiple sclerosis.

We prospectively recruited 15 patients with LGI1-AIE (CSF, *n* = 9; blood, *n* = 8) and CASPR2-AIE (CSF, *n* = 6; blood, *n* = 4) across three clinical centres (cohort 1), paying particular attention to immunotherapy naivety. As controls, we included prospectively recruited (*n* = 5) IIH patients and existing published data^21,32^ from MS and IIH patients as diseased controls (MS CSF, *n* = 15; MS PBMC, *n* = 5; IIH CSF, *n* = 18; IIH PBMC, *n* = 9; Supplementary Table 1). To avoid pre-analytical bias and cell loss, we directly *ex vivo* analysed fresh CSF and blood cells acquired in parallel using scRNA-seq (Fig. 1A). Sample processing was standardized between centres to minimize systematic technical bias (see the ‘Materials and methods’ section). Clinical, MRI and CSF features of patients were representative of the known phenotype of LGI1- and CASPR2-AIE, and all patients had core symptoms of limbic encephalitis (Supplementary Table 1). In brief, three of nine LGI1-AIE and zero of seven CASPR2-AIE patients were female; the average age of the combined group was 63 years (95% confidence interval, 60–66 weeks) and the median time from onset of symptoms was 17 weeks (95% confidence interval, 16–55 weeks; Supplementary Table 1). Basic CSF analyses (white blood cell numbers, protein and lactate) did not differ significantly between LGI1- and CASPR2-AIE (Supplementary Fig. 1A–C), in accordance with previous studies.^9,33^ The median CSF cell count was 5/µl (95% confidence interval, 3–8/µl), and all patients had <500 erythrocytes/µl CSF.

### CSF plasma cell expansion is a hallmark of LGI1-AIE and CASPR2-AIE

Initially, we analysed CSF cells and PBMCs by scRNA-seq in an unbiased fashion. We merged all scRNA-seq data from CSF and PBMCs of AIE, MS and IIH patients, which encompassed 328 547 total single-cell transcriptomes (CSF, 154 177; blood, 174 370), with a mean ± standard error of the mean of 4440 ± 412 cells per sample and 1218 ± 0.83 genes detected per cell (Supplementary Table 2). Single-cell transcriptomes are henceforth termed ‘cells’ for simplicity.

We performed cell clustering (Fig. 1B and Supplementary Fig. 1D) and semi-automatically annotated the resulting 11 cell clusters (Supplementary Table 3 and Supplementary Fig. 1E). Across patients and controls, T cells (CD4^+^ > CD8^+^) and myeloid-lineage cells dominated in CSF, as described previously.^32^ However, when comparing LGI1- and CASPR2-AIE patients with the IIH and MS controls, we found a pronounced increase of CSF antibody-secreting B lymphocytes (ASCs expressing *CD19*, *CD38* and *IGHG4*) both in LGI1-AIE and in CASPR2-AIE patients compared with the control groups (Fig. 1C–F). Relative to MS (but not IIH) controls, we also observed an increase in monocytes and myeloid dendritic cells and a decrease in naive and memory B cells in LGI1- and CASPR2-AIE CSF (Fig. 1E and F). Other proportions of CSF and PBMC cell lineages were not significantly and consistently altered between encephalitis variants at this accuracy level (Fig. 1C–F and Supplementary Fig. 1F–K). In summary, the main and consistent finding was the CSF expansion of ASCs in AIE patients.

We next sought to confirm these transcriptomics-based observations using flow cytometry. Therefore, we retrospectively identified an independent cohort of treatment-naive LGI1-AIE (*n* = 7) and CASPR2-AIE (*n* = 7) patients (Fig. 1A, cohort 2, three patients also in cohort 1) whose CSF cells had been analysed by multicolour flow cytometry as part of the routine clinical work-up (see the ‘Materials and methods’ section) (Supplementary Table 1 and Supplementary Fig. 2A). Patients with MS (*n* = 13) and functional neurological disorders (*n* = 14) served as inflammatory and non-inflammatory controls, respectively, and were propensity matched in age and sex (Supplementary Table 1). Basic clinical and CSF parameters of cohort 2 were similar to cohort 1 and replicated the known features of LGI1-/CASPR2-AIE patients (Supplementary Table 1 and Supplementary Fig. 2B–D). In this retrospective CSF flow cytometry dataset, we confirmed the expansion of plasma cells (LGI1-/CASPR2-AIE, CD3^−^CD19^+^CD138^+^) in the CSF of AIE patients compared with non-inflammatory controls (Fig. 1G and Supplementary Fig. 2E–J).

### CSF plasmablasts and plasma cells are clonally expanded, detect autoantigen and express IgG2 and IgG4 isotypes

**Figure 2:**
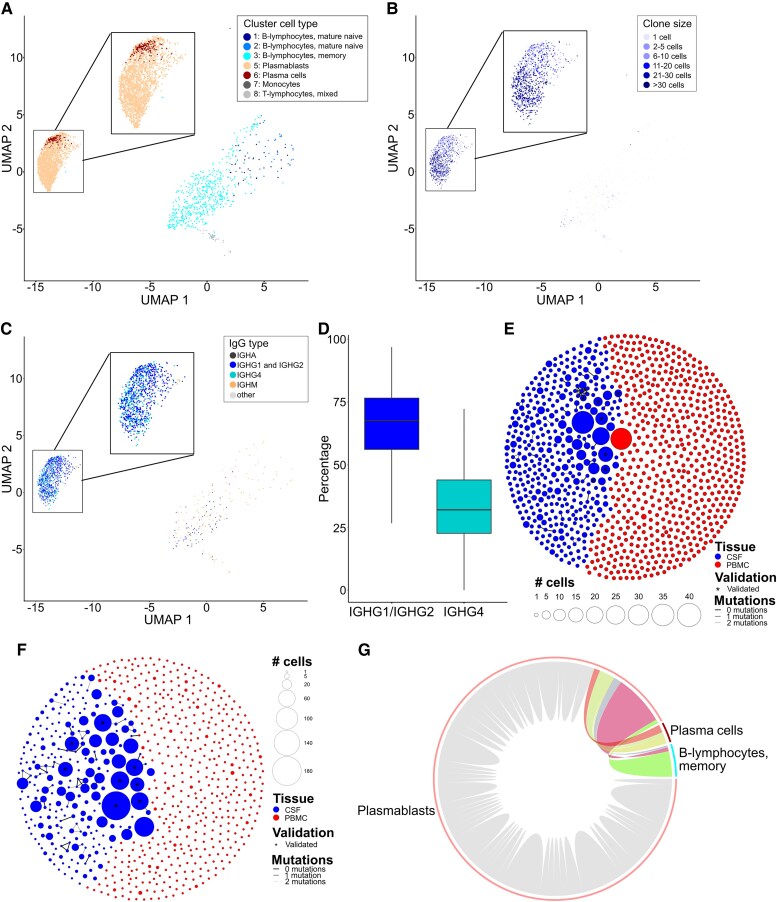
**B-lineage cells in CSF are preferentially plasmablasts, clonally expanded, and express IgG1/2 and IgG4 heavy chains in LGI1-/CASPR2-AIE.** Single-cell transcriptomes of all B-cell clusters (from Fig. 1B) in CSF and PBMCs from LGI1, CASPR2, IIH and MS patients were subclustered and analysed. (**A**) UMAP plot with B-lineage subclusters of CSF cells from AIE patients. Cluster 4 contains only PBMCs and is therefore not shown here. Expressions of selected marker genes are shown in Supplementary Fig. 3B and C. Cluster 8 is composed of ‘contaminating’ T cells. (**B**) UMAP plot illustrating the distribution of B-cell clone sizes in CSF from AIE patients. (**C**) UMAP depicting the immunoglobulin subtype transcribed by CSF cells from AIE patients. (**D**) Comparison of the frequency of transcribed immunoglobulin heavy chains (IGHG1 or IGHG2 versus IGHG4) in antibody-secreting B lymphocytes (ASCs) from CSF in all AIE patients. Only samples with >30 cells were considered. Significance was tested with a Mann–Whitney U-test. (**E** and **F**) Visual representation of B-cell receptor (BCR) clones (and clonotypes connected by lines) in the CSF (blue) and PBMCs (red) of two representative patients with LGI1-AIE (**E**) and CASPR2-AIE (**F**). Each dot indicates the amount of identical BCRs belonging to one clone. The area of each dot is proportional to the clone size. Clonotypes (connected by lines) were defined as identical VDJ gene segments, identical CDR3 length and a CDR3 nucleotide sequence with two or fewer mismatches. Asterisks mark clones that were verified experimentally as autoantigen specific. (**G**) Circos plot highlighting BCR clones composed of mixtures of plasmablasts, plasma cells and memory B cells. The line thickness indicates the number of cells. Clones consisting of only one cell type are marked in grey. AIE = autoimmune encephalitis; PBMC = peripheral blood mononuclear cell; UMAP = uniform manifold approximation and projection; VDJ = variable–diversity–joining.

Considering the strong signal on plasma cell expansion, we further characterized the CSF B-lineage cells in LGI1- and CASPR2-AIE. We subclustered all single-cell transcriptomes annotated as B lineage from the merged PBMC/CSF scRNA-seq dataset. CSF B-lineage cells in AIE patients were dominantly plasmablasts and less frequently plasma cells (Fig. 2A), whereas the majority of B-lineage cells in IIH and MS disease controls were mature and memory B cells (Supplementary Fig. 3A), as supported by their respective expression profile (Supplementary Fig. 3B and C and Supplementary Table 4). Notably, clonally expanded cells mostly resided in the plasmablast cluster of AIE patients (Fig. 2B). CSF ACSs (plasma cells and plasmablasts) in AIE patients transcribed not only *IGHG4* but also *IGHG1*/*IGHG2* in comparable frequencies (Fig. 2C and D and Supplementary Fig. 3D) independent of whether the ASC was annotated as a plasmablast or a plasma cell (Supplementary Fig. 3E). Analysing transcripts and pathways relevant for therapeutic approaches, ASCs transcribed reduced (compared with memory B cells) but still detectable amounts of *CD19*, *IL6R* and the accessory B-cell receptor component *CD79B* (Supplementary Fig. 3F). They transcribed almost no *MS4A1*/CD20 and showed downregulation (compared with plasma cells in blood) of the proteasome pathway, targeted by bortezomib, a drug currently investigated in AIE^34^ (Supplementary Fig. 3G and H). Between LGI1- and CASPR2-AIE, cell type and isotype abundance did not differ significantly (Supplementary Fig. 3D).

Next, we visualized the entire CSF and blood B-cell repertoires for all patients. Exemplary graphs are shown for one LGI1- and one CASPR2-AIE patient (Fig. 2E and F). As described previously in smaller cohorts,^8,11^ we confirmed a highly restricted, clonally expanded, somatically strongly hypermutated B-cell repertoire in the CSF of CASPR2- and LGI1-AIE patients (Supplementary Fig. 4). Most expanded clones consisted of plasma cells and plasmablasts, and some CSF clones also contained class-switched memory B cells (Fig. 2G). There were only few shared clones between CSF and blood (median <5% in LG1 and CASPR2), and these were primarily small clones of fewer than five cells.

In summary, LGI1- and CASPR2-AIE patients harbour clonally expanded CD19-, IL6-receptor- and B-cell receptor-expressing plasmablasts in their CSF, which exclusively transcribe autoantigen-specific IgG1, IgG2 and IgG4 antibodies and show signs of affinity maturation occurring mostly (but not exclusively) outside of the CSF compartment.

### AIE-specific subgroups of CD4 and CD8 central memory T cells in CSF are clonally expanded and activated

**Figure 3:**
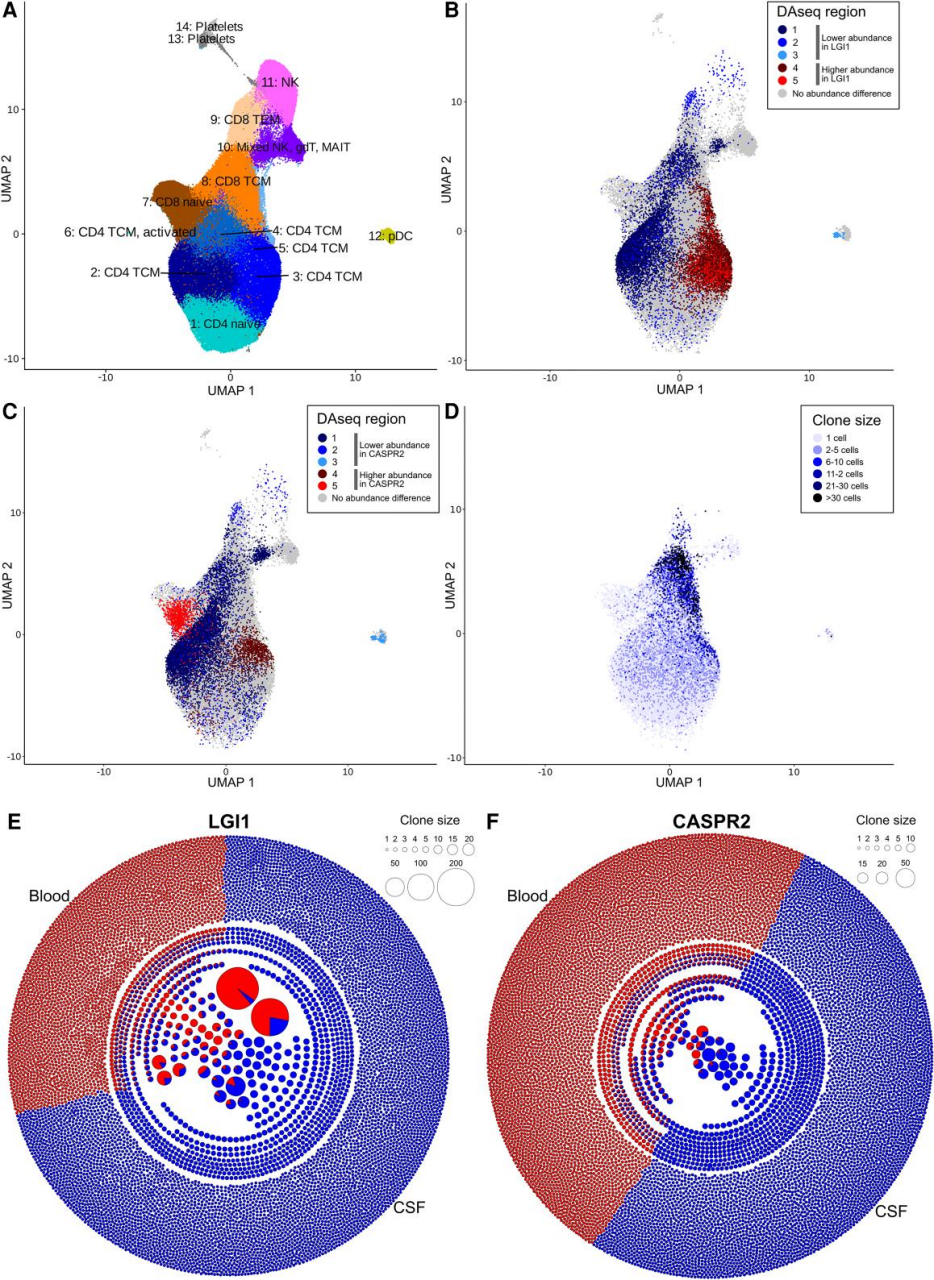
**T-cell repertoires in CSF of LGI1-/CASPR2-AIE patients show differential changes preferentially in the CD4 TCM clusters, clonal expansion of activated CD4 TCM and CD8 TCM clusters, and blood–CSF-spanning T-cell clones.** (**A**) UMAP plot based on the T-cell subclustering (of all T-cell clusters from Fig. 1B) of all PBMCs and CSF cells. (**B** and **C**) Unbiased, cluster-free analysis of differential T-cell abundance was analysed in a pairwise fashion using the DAseq tool and visualized in shades of red for increases and shades of blue for decreases; comparison of LGI1 and IIH CSF T cells (**B**) and CASPR2 and IIH CSF T cells (**C**). (**D**) T-cell clone sizes in CSF from AIE patients projected onto the UMAP plot. (**E** and **F**) Visual representation of T-cell receptor clones in CSF (blue) and PBMC (red) of two representative patients with LGI1-AIE (**E**) and CASPR2-AIE (**F**). In the network, each dot indicates one clone (identical VDJ genes and CDR3 regions). The area is proportional to the clone size. The pie charts indicate the percentage of CSF cells and PBMCs within each clone. AIE = autoimmune encephalitis; IIH = idiopathic intracranial hypertension; MAIT = mucosal-associated invariant T cell; NK = natural killer cell; PBMC = peripheral blood mononuclear cell; TCM = central memory T cell; UMAP = uniform manifold approximation and projection; VDJ = variable–diversity–joining.

Therefore, we next characterized T cells in the CSF of LGI1- and CASPR2-AIE. Initially, we subclustered and annotated all cells identified as T cells. As described,^32^ T cells spanned a transcriptional gradient ranging from naive towards memory phenotypes (Fig. 3A, Supplementary Fig. 5A and Supplementary Table 5) rather than forming distinct subclusters. T-cell subclusters in CSF were not consistently different between AIE patients and controls (Supplementary Fig. 5B). However, cell–cell interaction networks indicated higher interaction strength of CD8 T-effector memory with CD4 central memory T cells (TCM) and B lymphocytes in AIE (Supplementary Fig. 5C), and a cluster approach might have missed within-subcluster changes in abundance. Therefore, we adopted a cluster-free approach to identify subgroups with different abundance between AIE and IIH/MS samples.^35^ Indeed, we identified differentially regulated, distinct subgroups of CD4 TCM in the CSF of AIE patients compared with IIH and MS controls (Fig. 3B and C and Supplementary Fig. 6A–F). The relative changes were localized compared with IIH patients (Fig. 3B and C) and rather widespread compared with MS (Supplementary Fig. 6A and B). AIE subtypes were similar, yet CASPR2-AIE patients additionally showed a differential increase in abundance of naive CD8 T cells (Supplementary Fig. 6C–F). Considering that the main changes were observed within the CD4 TCM compartment, we queried the differentially abundant CD4 TCM cells for their specific gene set enrichment. CD4 TCM cells increased in LGI1- and CASPR2-AIE CSF preferentially upregulated stress and protein degradation/processing pathways, whereas the cells increased in CSF of control patients showed more typical T-cell gene sets (Supplementary Fig. 6G–J).

We also observed clonally expanded T cells preferentially in the CSF of patients with AIE. Clonally expanded T cells in general were localized predominantly within certain CD8 and CD4 T central memory subclusters (Fig. 3D and Supplementary Fig. 7A). Gene set enrichment analyses showed active differentiation and cell survival pathways (Supplementary Fig. 7B and C). We next performed detailed T-cell receptor (TCR) repertoire analysis. These are shown for two representative patients (Fig. 3E and F). Overall, these visualizations demonstrate the clonal expansion (Supplementary Fig. 7D) and restriction of the T-cell repertoire in CSF of AIE patients (Supplementary Fig. 7E). Multiple large clones spanned CSF and blood compartments (Fig. 3E and F and Supplementary Fig. 7F and G).

In summary, specific CSF T-cell subsets in LGI1-/CASPR2-AIE were clonally expanded, activated, located in the CD4 and CD8 TCM compartment, partly overlapping with blood clones, and some showed transcriptional changes associated with oxidative and metabolic stress.

### Innate-like T cells are significantly reduced in the CSF and peripheral blood in LGI1- and CASPR2-AIE

**Figure 4:**
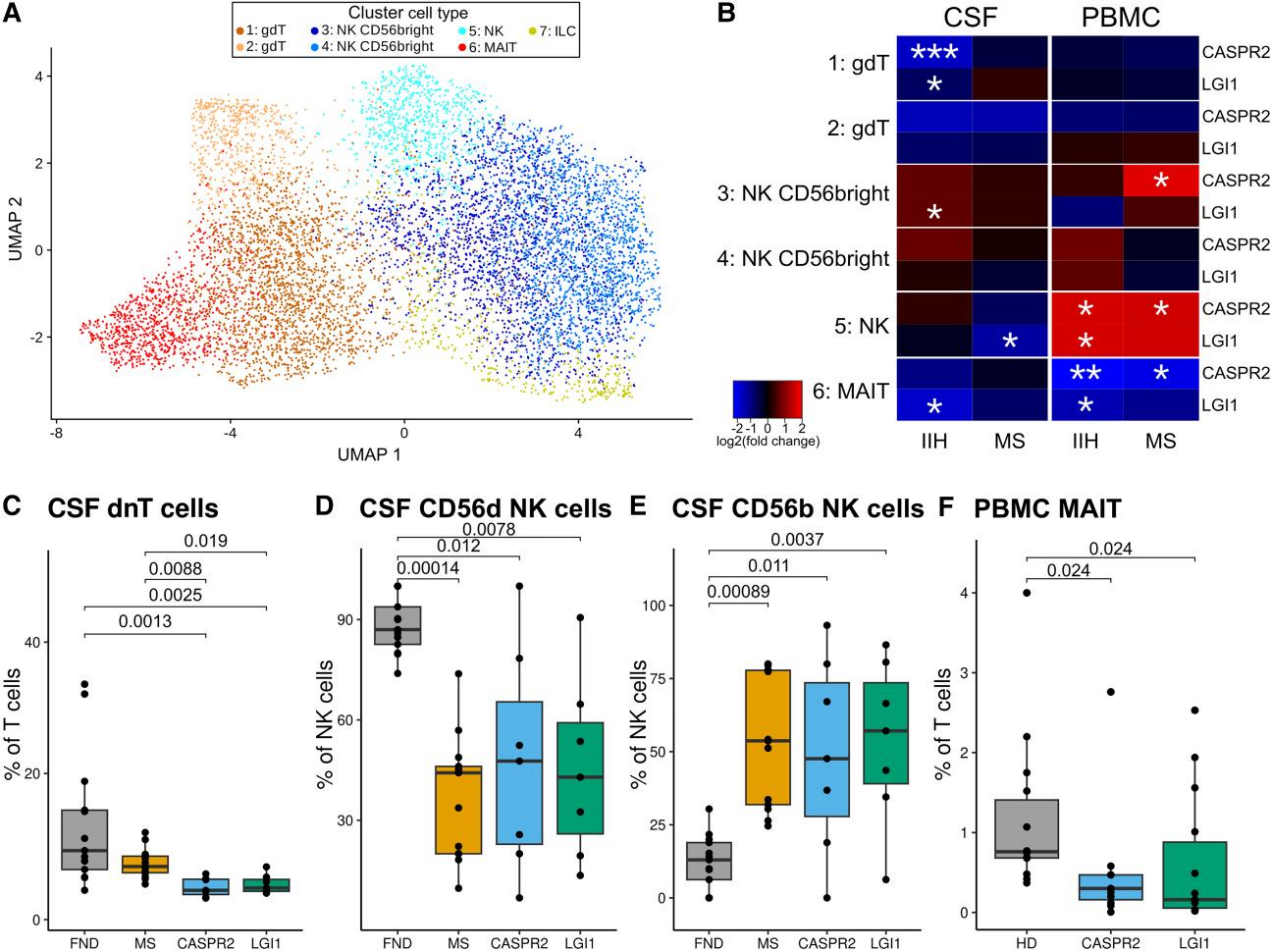
**Patients with LGI1-/CASPR2-AIE show trans-compartment loss of innate-like MAIT cells and shifts in the NK cell subpopulations.** (**A**) UMAP showing the subclustering of the mixed T-cell cluster (cluster 10, Fig. 3). The expression levels of marker genes for each cell type shown are visualized in Supplementary Fig. 8A. (**B**) Heat map comparing selected cell-type abundances (of the subclustering shown in Fig. 4A) between AIE patients, IIH and MS controls across CSF and PBMC compartments. Colours indicate an increase (red) or decrease (blue), and asterisks indicate significance (Supplementary Fig. 8B and C). (**C**–**E**) Flow cytometry validation: cell proportions of dnTc (CD3^+^CD4^−^CD8^−^) (**C**), CD56dim NK cells (CD3^−^CD56^dim^) (**D**) CD56bright NK cells (**E**), in the CSF (cohort 2). (**F**) Cell proportion of MAIT cells in PBMCs in another independent flow cytometry cohort (cohort 3). Frequency respective to parent gate. Gating schemes are shown in Supplementary Figs 2 (cohort 2) and 9 (cohort 3). AIE = autoimmune encephalitis; FND = functional neurological disorders; IIH = idiopathic intracranial hypertension; MAIT = mucosal-associated invariant T cell; MS = multiple sclerosis; NK = natural killer cell; PBMC = peripheral blood mononuclear cell.

The cluster-free approach to evaluate T-cell abundance also indicated differential regulation in subgroups of a mixed cluster of unconventional, invariant T/NK cells composed of γδ T cells, NK cells and MAIT cells (Fig. 3B and C and Supplementary Fig. 6A–F). Accordingly, we performed subclustering of this mixed cohort and analysed differential abundance (Fig. 4A and Supplementary Fig. 8A). We observed a significant reduction of cells annotated as MAIT (e.g. *CXCR6*, *KLRB1*/*CD161* and *ZBTB16*; Fig. 4B) in LGI1-AIE and γδ T cells (cluster named gdT; e.g. *TRDC*) in the CSF of all AIE patient cells compared with IIH controls (Fig. 4B). Intriguingly, we observed an even stronger and consistent reduction in MAIT cells in the blood of all AIE patients relative to IIH and MS patients, together with an opposing increase in NK cells (Fig. 4B and Supplementary Fig. 8B and C).

Although the marker profile used in our CSF flow cytometry data in cohort 2 did not allow for a specific quantification of MAIT and γδ T cells, we could show a significant decrease of double-negative T cells (dnTc) (Fig. 4C) in the CSF of LGI1- and CASPR2-AIE patients, supporting a shift in unconventional T cells in CASPR2- and LGI1-AIE. We could also demonstrate a shift from CD3^−^CD56^+dim^ NK cells (Fig. 4D) to less cytotoxic CD56bright NK-cell phenotypes in CASPR2-/LGI1-AIE CSF compared with IIH but not MS control patients (Fig. 4E).

Considering the consistent and specific reductions of MAIT cell frequencies in the systemic compartment in the scRNA-seq cohort 1, we next analysed a third independent cohort (cohort 3; Supplementary Table 1). We specifically characterized MAIT cells by flow cytometry using available cryo-preserved PBMCs from patients with LGI1-AIE (*n* = 14), CASPR2-AIE (*n* = 11) and matched healthy controls (*n* = 14). This cohort also featured clinical and CSF features in accordance with the disease (Supplementary Fig. 9A–C and Supplementary Table 1). We confirmed a significant decrease of blood MAIT cells in LGI1- and CASPR2-AIE compared with controls (Fig. 4F). As in our scRNA-seq cohort 1, we did not observe significant changes in γδ T cells in blood (Supplementary Fig. 9D and E). In summary, a reduction of innate-like T cells, specifically MAIT cells in the CSF and blood, in addition to a shift in NK-cell phenotypes (towards less cytotoxic CD56bright in the CSF) of patients with LGI1-AIE and CASPR2-AIE is part of the distinct invariant T-cell and innate immune-cell profile changes of this disease.

### MAIT cells infiltrate the brain in AIE and suppress peripheral anti-neuronal humoral autoimmunity

**Figure 5:**
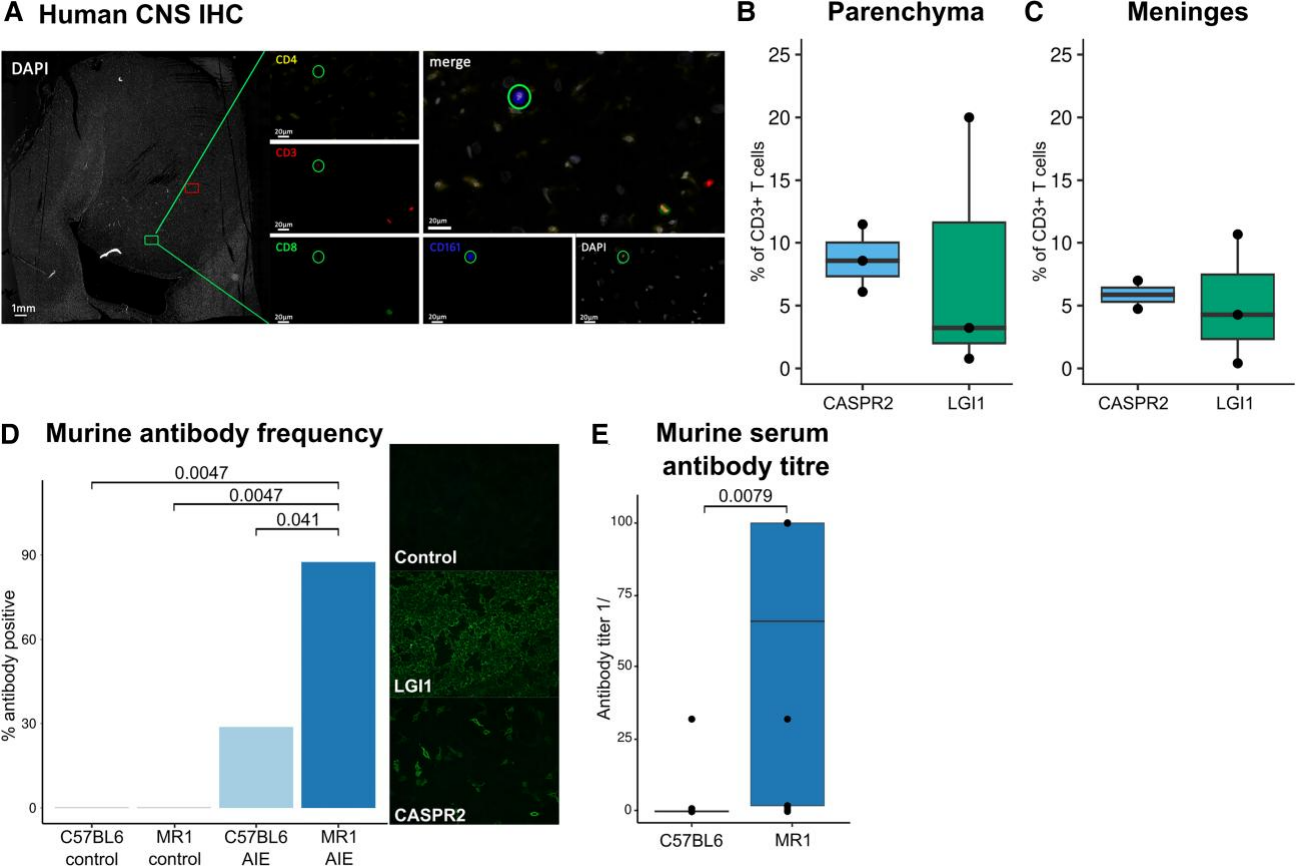
**MAIT cells are present in the brain of patients with CASPR2-AIE and LGI1-AIE, and their absence leads to reduced presence of autoantibodies in a murine immunization model.** (**A**) Multiplexed immunofluorescence (mIF) staining of post-mortem CASPR2-AIE brain tissue, showing CD3 (red), CD8 (green), DAPI (white), CD4 (yellow) and CD161 (blue). Scale bars = 1 mm in overview; 20 μm in subpanels. (**B** and **C**) Box plots representing CD3^+^CD161^+^CD4-gdTc-CD8^+/−^ T cells as a percentage of CD3^+^ cells in the parenchyma (**B**) and meninges (**C**) of three human CASPR2- and three LGI1-AIE patients as shown in multiplex immunohistochemistry. (**D** and **E**) Murine immunization model using full-length LGI1/CASPR2 protein immunization (AIE) versus sham immunizations (control) of MAIT-deficient mice (MR1) and littermates (C57BL6). Serum was analysed on Day 28 for LGI1/CASPR2 antibody positivity using a cell-based assay, and the end point titre was determined. (**D**) Bar plot depicting percentage of antibody-positive mice. (**E**) Box plot showing antibody titre with respect to their disease group. Statistical significance was determined by Fisher’s exact test in **D** and by Wilcoxon rank test in **E**. AIE = autoimmune encephalitis; IHC = immunohistochemistry; MAIT = mucosal-associated invariant T cell.

Having identified a reduction of MAIT cells in the CSF and blood of AIE patients, we asked whether these cells egress into the brain as one possible explanation for their dwindling numbers in CSF and blood. Indeed, in addition to their function in suppressing peripheral autoimmunity, MAIT cells have been shown to accumulate in the inflamed organs in some autoimmune diseases.^36,37^ We therefore next investigated whether similar mechanisms might apply to AIE. We queried the available rare autopsy materials of three LGI1-AIE and three CASPR2-AIE patients for signs of significant T-cell infiltration in the parenchyma and meninges. Of these cases, three CASPR2-AIE and three LGI1-AIE patients showed signs of T-cell infiltration, which were investigated further by multicolour immunohistochemistry (Fig. 5A and Supplementary Table 6). We identified CD3^+^CD161^+^TCRγδ^−^CD4^−^ cells that resemble MAIT cells in the meninges (Fig. 5B) and parenchyma (Fig. 5C) (hippocampus and uncus; Fig. 5A); regions predominantly affected by the disease.

To provide arguments for causality over association of this MAIT-cell phenotype, we performed murine experiments to confirm functionally that MAIT cells influence systemic humoral anti-LGI1/anti-CASPR2 autoimmunity. We actively immunized mice with the recombinant extracellular portion of LGI1 and CASPR2 proteins (see the ‘Materials and methods’ section; Supplementary Fig. 10A), adopting an immunization scheme previously used in modelling anti-NMDAR-AIE.^38^ After 28 days, 29% (two of seven) of C57BL6 mice showed detectable LGI1/CASPR2 autoantibodies in serum (Fig. 5B). Concurrently, we immunized littermates genetically deficient in the invariant MR1 antigen-presenting molecule that consequently lack MAIT cells.^39^ Immunization (five CASPR2 and three LGI1) induced antineuronal serum antibodies in 88% (seven of eight) of MR1-deficient mice (Fig. 5D). Anti-LGI1/anti-CASPR2 antibodies remained undetectable in unimmunized mice from both genotypes (*n* = 10; Fig. 5D). In addition to antibody prevalence, antibody titres were also significantly higher in immunized MR1-deficient mice than in immunized C57BL6 controls (Fig. 5E). Using an active immunization approach modelling induction of autoimmunity, we thus demonstrated that loss of MAIT cells facilitated peripheral anti-neuronal humoral autoimmunity. Of note, we did not observe CNS infiltration and clinical signs of AIE in a subset of these mice (MR1 AIE, *n* = 6; C57BL6 AIE, *n* = 4; MR1 control, *n* = 5; C57BL6 control, *n* = 5) using flow cytometry of gross CNS-infiltrating leucocytes (Supplementary Fig. 10B) and behavioural testing (three-chamber test, open field test, learning and memory novel object recognition; Supplementary Fig. 10C). This indicates that peripheral anti-LGI1/anti-CASPR2 antibodies do not suffice to induce clinically and analytically tangible AIE-like disease in mice. This could be attributable to insufficient CNS barrier breach, absence of a required ‘second hit’ or low MAIT cell numbers in mice compared with humans.^39,40^

In summary, our findings suggest that MAIT cells might participate in AIE pathomechanisms by peripherally suppressing anti-neuronal autoantibody production and intrathecally promoting tissue damage, similar to what has been described across diverse other autoimmune conditions.^36,37,41-43^

## Discussion

We here provide a deep, comprehensive characterization of autoimmunity in a large and representative cohort of untreated patients with LGI1- and CASPR2-AIE, focusing on B- and T-cell interaction and shared components between CSF and blood. We made several main observations. First, we confirmed prior observations^8,11^ in a significantly larger and thus potentially more representative cohort that the single most dominant cellular change in the CSF in LGI1-/CASPR2-AIE was an expansion of ASCs, predominantly plasmablasts. These ASCs showed a high degree of clonal expansion, somatic hypermutation and antigen specificity. We could also confirm^8^ that a low degree of intraclonotype mutations indicated that affinity maturation occurred mostly outside of the CNS. However, in our cohort, equal amounts of plasma cells/plasmablasts transcribed IgG1/2 compared with IgG4. Second, we identified distinct qualitative transcriptional changes in the (quantitatively unchanged) conventional T-cell compartment in the CSF, most notably in the CD4 TCM compartment. Activated CD4 TCM and CD8 TCM cells showed clonal expansion with clones spanning CSF and blood, clonal restriction, T-cell activation and signs of metabolic stress. Third, we unexpectedly identified a cross-compartment shift in NK cell phenotypes and reduction of innate-like T cells, especially MAIT cells. We also showed their presence within affected brain tissue in patients with CASPR2-AIE. In a novel animal immunization paradigm, the absence of MAIT cells led to an increased seroprevalence and titre of the respective anti-neuronal antibodies. We thus propose a model where loss of peripheral innate-like regulatory mechanisms disinhibits proliferation of B cells (possibly via their effect on cognate T cells), which then drive intrathecal production of anti-neuronal autoantibodies.

Expanding previous lines of evidence in a larger cohort of 15 patients, we confirmed the presence of highly expanded clones of ASCs in the CSF of patients with LGI1- and CASPR2-AIE with a high frequency of antigen-specific clones, as previously shown.^8^ The degree of clonal restriction, expansion and antigen specificity sharply contrasted with the absence of increased CSF white blood cells and CSF-restricted oligoclonal bands. All examined strongly expanded clones showed antigen specificity, indicating a highly selective ongoing expansion of CSF B cells. In addition, we observed the transcription of approximately equal proportions of IgG4 and non-IgG4 heavy chains by CSF ASCs. Indeed, neuronal loss has been hypothesized to be caused by complement-fixing IgG1–3 isotype LGI1 autoantibodies,^44,45^ which makes this observation relevant and the respective ASCs a promising therapeutic target. Our observation on a cellular level contrasts with serological studies suggesting that LGI1- and CASPR2-AIE CSF autoantibodies are mostly IgG4.^46,47^ One possible explanation warranting confirmation would be that IgG4-producing ASCs translate more transcripts into antibody protein than their non-IgG4 counterparts.

The primary suspects potentially driving, orchestrating and propagating this B-cell phenotype were T cells. Indeed, the HLA class II restriction observed in these diseases can be explained only by a relevant T-cell participation in disease pathogenesis. Our data now provide new compelling evidence of a dysregulated conventional T-cell compartment in the CSF with significant systemic cross-talk. Indeed, the CD4^+^ TCM cells in CSF were antigen-experienced, proliferating and expressing activation markers. T cells with B-cell-helping capabilities (Tph) were not identified in high numbers, which, together with infrequent within-clone mutations detected in B cells, supports the notion^8^ that the T-cell-mediated affinity maturation in these diseases most probably occurs in the systemic compartment. However, we hypothesize that these clonally restricted CD4 TCM cells still play a significant role in driving the observed intrathecal B-cell expansion and possibly class-switching, because we still identified a large fraction of non-IgG4 ASCs in patients at the very early sampling time of our study.

Although it is clear that LGI1 antibodies cause reversible synaptic dysfunction, this does not exclude a relevant role of cytotoxic T cells, nor does the absence of a strong HLA class I restriction. Indeed, earlier histopathology in ‘VGKC-antibody-positive LE’, most of which were later found to be LGI1- or CASPR2-AIE, described cytotoxic CD8 T cells in the brain, albeit in a smaller percentage than in paraneoplastic cases with antibodies against intracellular antigens.^48^ In addition to the clear CD4 T-lymphocyte pattern, we could also identify a clonal expansion of CD8 TCM cells in the CSF of patients with LGI1- and CASPR2-AIE, which showed signs of activation. Taken together, an involvement of cytotoxic CD8 T cells and consecutive neuronal loss contributing to the often irreversible cognitive dysfunction in up to one-third of patients with LGI1-^47^ and CASPR2-AIE, especially in patients with delayed treatment, cannot be ruled out. Future work needs to confirm their presence at later disease stages and especially their antigen specificity and direct cytotoxic effects on neurons. Importantly, we identified significant cross-talk between the intrathecal and systemic T-cell compartments with significant amounts of shared clones between CSF and blood. Although the temporal dynamics of this cross-talk need to be elucidated, therapeutically addressing T cells using systemic approaches might thus also influence the intrathecal compartment.

Additionally, we observed a novel phenotype in the invariant T-cell compartments of patients with LGI1-/CASPR2-AIE. Surprisingly, these cells were found significantly less frequently in the CSF of patients, a phenotype also confirmed for MAIT cells in the blood. These invariant T cells detect conserved microbial metabolites and regulate mucosal immunity, yet have also been implicated in B-cell stimulation and autoimmunity.^49,50^ MAIT cells not only sense microbial patterns in tissue, but also locally shape adaptive antibody responses to infectious pathogens.^41^ Interestingly, the role of meninges in the regulation of CNS immune responses has gained renewed interest^51^ as an active neuroimmune interface. In accordance, dense perivascular B-cell and plasma-cell infiltrates have been observed in the meninges and Virchow–Robin spaces (together with CD4 T cells) of patients with LGI1-AIE^48^ (and NMDAR-AIE^52^). The meninges might, in fact, represent an integration point between the adaptive and innate immune system in AIE. In fact, we were able to identify T lymphocytes resembling MAIT cells in the brain parenchyma and meninges of six patients with LGI1-AIE or CASPR2-AIE. In the systemic immune compartment, MAIT cells might also contribute to the disease initiation by direct or indirect contact with danger signals in barrier tissue, e.g. the gut. Indeed, a peripheral loss of MAIT cells has been described consistently across autoimmune diseases,^36,37,43^ whereas select studies show an increase of MAIT cells in the targeted tissue, suggesting a compartment-specific function of MAIT cells.^37,53^ MAIT cells can promote humoral immunity^50,54-56^ by increasing the production and differentiation of plasmablasts in a feed-forward loop.^50,55^

Elucidating this connection between innate-like T cells and B cells necessitated immunization models emulating early components of autoimmunity. Models of passive immunization,^57-60^ including injection with CASPR2 and LGI1 antibodies,^57,58,61^ have previously been established successfully in AIE. Naturally, these models only allow study of the effector functions of the respective antibodies. T cell-stimulating approaches with vector-based intrathecal application have been published^62,63^ for limbic encephalitis but do not mimic the systemic antigen-based response. Recently, three similar active immunization methods have been demonstrated in NMDAR-AIE^38,64,65^ that could also show CNS inflammation and behavioural changes mimicking NMDAR-AIE. Thus, murine active immunization models in AIE remain challenging and model only parts of the disease. Although our model lacked clinical or cellular signs of CNS LGI1-/CASPR2 AIE, we were able to induce disease-defining antibodies.^4^ Our study might form the basis for mechanistic exploitation of such active immunization models in early disease steps of LGI1-/CASPR2-related disease and, specifically, of the role of MAIT cells in this context. The link between innate-like T cells and autoantibody-mediated encephalitis variants might identify exploitable therapeutic targets early in the pathogenic cascade of antibody-driven CNS autoimmunity. However, future studies need to confirm the tissue translocation and the functional link to B-cell proliferation in the CSF in model systems.

Finally, in our study, we provide one of the largest single-cell compendiums of CSF cells in any disease. Studies with similar design have accumulated 120 629 (*n* = 51 donors)^66^ and 216 723 single-cell transcriptomes (175 529 CSF and 41 194 blood; *n* = 57 donors) from MS patients and controls.^67^ Dementia-related CSF atlasing efforts have collected 22 625 (*n* = 22 donors) and 21 267 single-cell transcriptomes (*n* = 18 donors) from CSF.^68^ Our present dataset encompasses 328 547 total single-cell transcriptomes from 48 individuals (15 with AIE). Our AIE cohort is considerable (26 LGI1-AIE and 18 CASPR2-AIE patients across four cohorts), yet the low prevalence of LGI1-/CASPR2-AIE naturally complicated our ability to recruit even larger cohorts.

## Conclusion

Overall, our study provides a large-scale unbiased transcriptomic analysis with confirmation assays in independent cohorts in LGI1- and CASPR2-AIE. In the future, it will be crucial to extend this study to different AIE variants, to longitudinal studies of patients receiving treatment (e.g. B cell-depleting treatment) and to consider further trans-compartment analyses (e.g. CSF–lymph nodes). Our study also provides potential for mechanistic understanding and diagnostic and therapeutic markers to benefit personalized treatments in autoantibody-mediated brain diseases.

## Supplementary Material

awaf096_Supplementary_Data

## Data Availability

The sequencing raw data and processed data will be made available in the European Genome-Phenome Archive upon reasonable request and after material transfer agreement to regulate data protection of potentially re-identifiable genetic data.

## Appendix 1

### EMC AIE Study group

Full details are provided in the Supplementary material.

Juna M. de Vries, Mariska M. P. Nagtzaam, Suzanne C. Franken, Yvette S. Crijnen, Juliette Brenner, Robin W. van Steenhoven, Jeroen Kerstens, Marienke A. A. M. de Bruijn, Anna E. M. Bastiaansen, Remco M. Hoogenboezem, Sharon Veenbergen and Peter A. E. Sillevis Smitt.
