# Supplementary material for: Activated αβ T and reduced mucosa-associated invariant T cells in LGI1- and CASPR2-encephalitis
Source: Brain. 2025 Mar 17;148(9):3170–83. doi: 10.1093/brain/awaf096 (PMC12404778; doi:10.1093/brain/awaf096)
Supplement: awaf096_Supplementary_Data [file awaf096_supplementary_data.zip › brain-2024-00707-File007.pdf]

Figure S1:

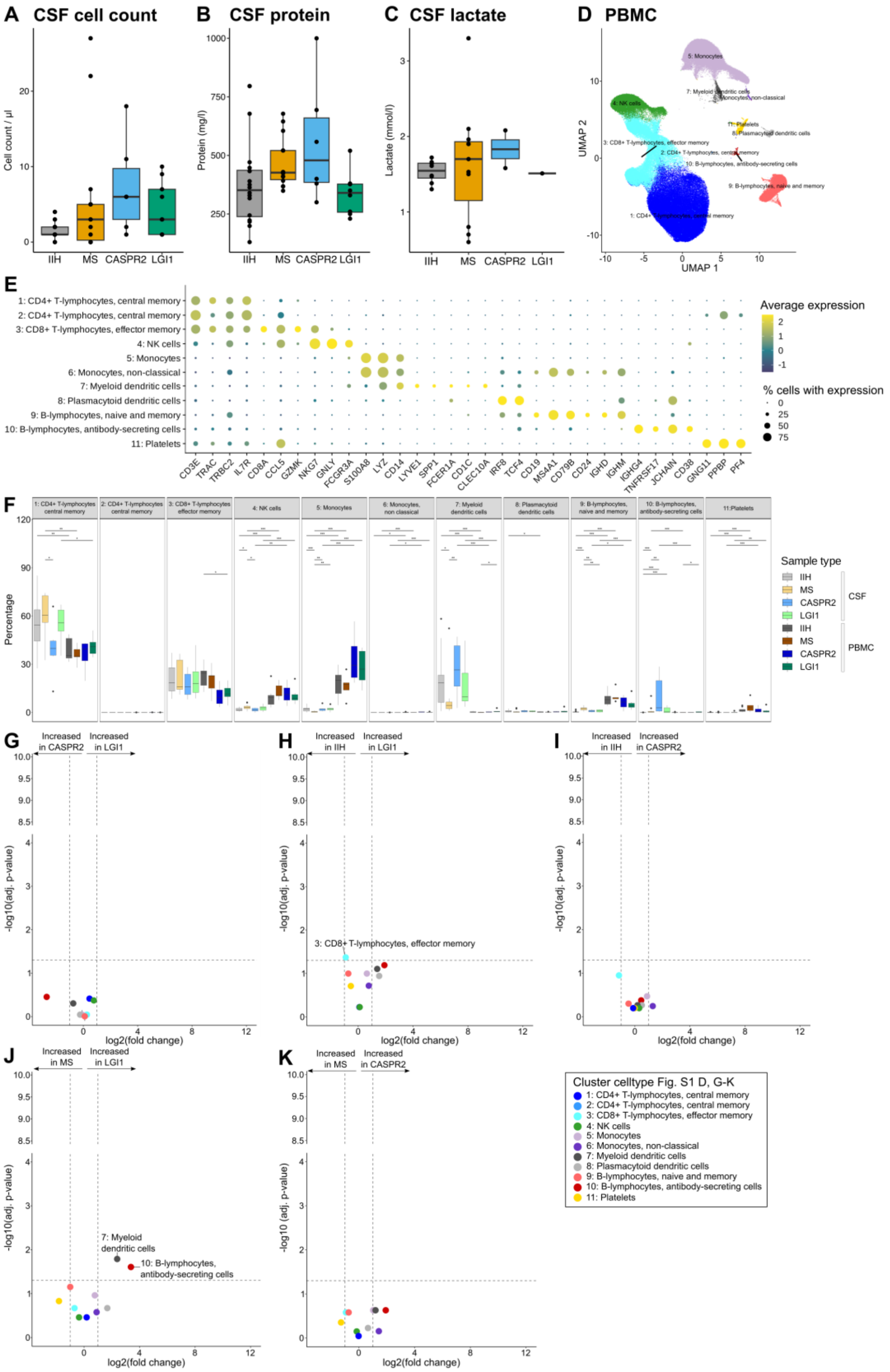
A CSF cell count
B CSF protein
C CSF lactate
D PBMC
E
F
G
H
I
J
K
Cell count / µl
Protein (mg/l)
Lactate (mmol/l)
UMAP 1
UMAP 2
IIH
MS
CASPR2
LGI1
Average expression
% cells with expression
Percentage
Sample type
CSF
PBMC
-log10(adj. p-value)
log2(fold change)
Increased in CASPR2
Increased in LGI1
Increased in IIH
Increased in MS
3: CD8+ T-lymphocytes, effector memory
7: Myeloid dendritic cells
10: B-lymphocytes, antibody-secreting cells
Cluster celltype Fig. S1 D, G-K
1: CD4+ T-lymphocytes, central memory
2: CD4+ T-lymphocytes, central memory
3: CD8+ T-lymphocytes, effector memory
4: NK cells
5: Monocytes
6: Monocytes, non-classical
7: Myeloid dendritic cells
8: Plasmacytoid dendritic cells
9: B-lymphocytes, naive and memory
10: B-lymphocytes, antibody-secreting cells
11: Platelets

Fig. S1 Basic CSF features, scRNAseq based cell cluster annotation and frequencies in CSF and PBMCs of patients with LGI1-/CASPR2-AIE and controls (cohort #1)
(A-C) Basic CSF parameters of cohort #1 are depicted as boxplots across IIH, MS, CASPR2-AIE, and LGI1-AIE. (D) UMAP plot showing the distribution of all cell type clusters for PBMCs. (E) Gene expression level of marker genes for all cell subtypes in the main clustering. (F) Boxplot comparing the cell type distribution for all sample groups. P-values were determined with the tool propeller for all comparisons within each tissue and for each sample type between tissues. (G) Comparison of cell type cluster abundances between CASPR2 und LGI1 in CSF. (H-K) Comparison of cell type cluster abundances in PBMC.

Figure S2:

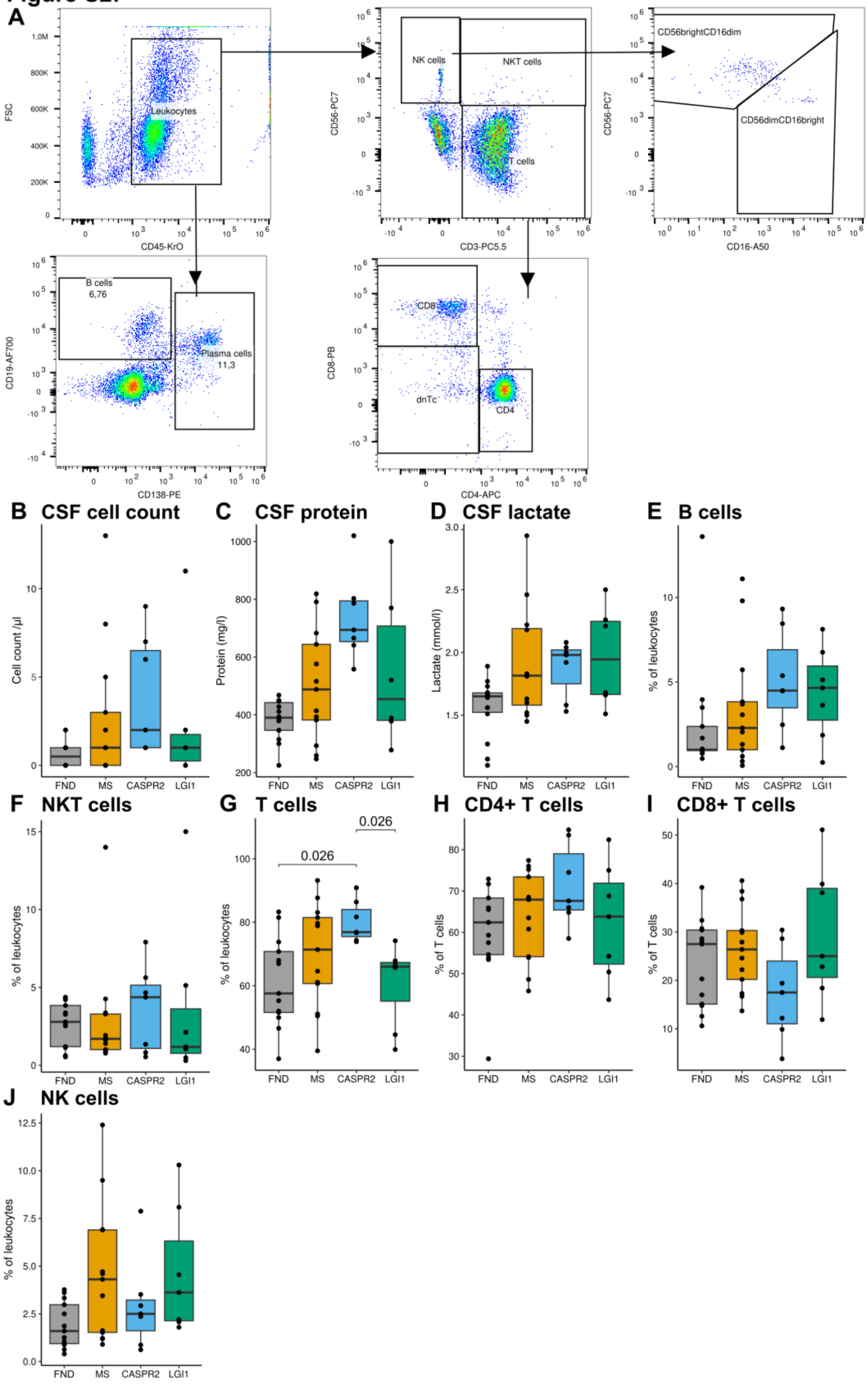
A
Leukocytes
FSC
CD45-KrO
NK cells
NKT cells
T cells
CD56-PC7
CD3-PC5.5
CD56brightCD16dim
CD56dimCD16bright
CD16-A50
B cells
6,76
Plasma cells
11,3
CD19-AF700
CD138-PE
CD8
dnTc
CD4
CD8-PB
CD4-APC
B CSF cell count
Cell count /µl
C CSF protein
Protein (mg/l)
D CSF lactate
Lactate (mmol/l)
E B cells
% of leukocytes
F NKT cells
G T cells
0.026
0.026
H CD4+ T cells
% of T cells
I CD8+ T cells
J NK cells
FND
MS
CASPR2
LGI1

Fig. S2 Flow cytometry gating strategy, basic CSF features, and flow cytometry based cell frequencies in CSF of patients with LGI1-/CASPR2-AIE and controls (cohort #2)

(A) Flow cytometry representative gating is shown in pseudocolor plots. Blood and CSF cells were gated on forward scatter and side scatter followed by marker genes as shown. (B-D) Clinical CSF parameters in cohort #2. (E-J) FACS data of cohort #2. Immune cell frequencies are displayed as percentages of their parent gate (A). The statistical significance was calculated with the Kruskal-Wallis test with post-hoc Dunn's test. The p values were adjusted with the Benjamini-Hochberg's method.

Figure S3:

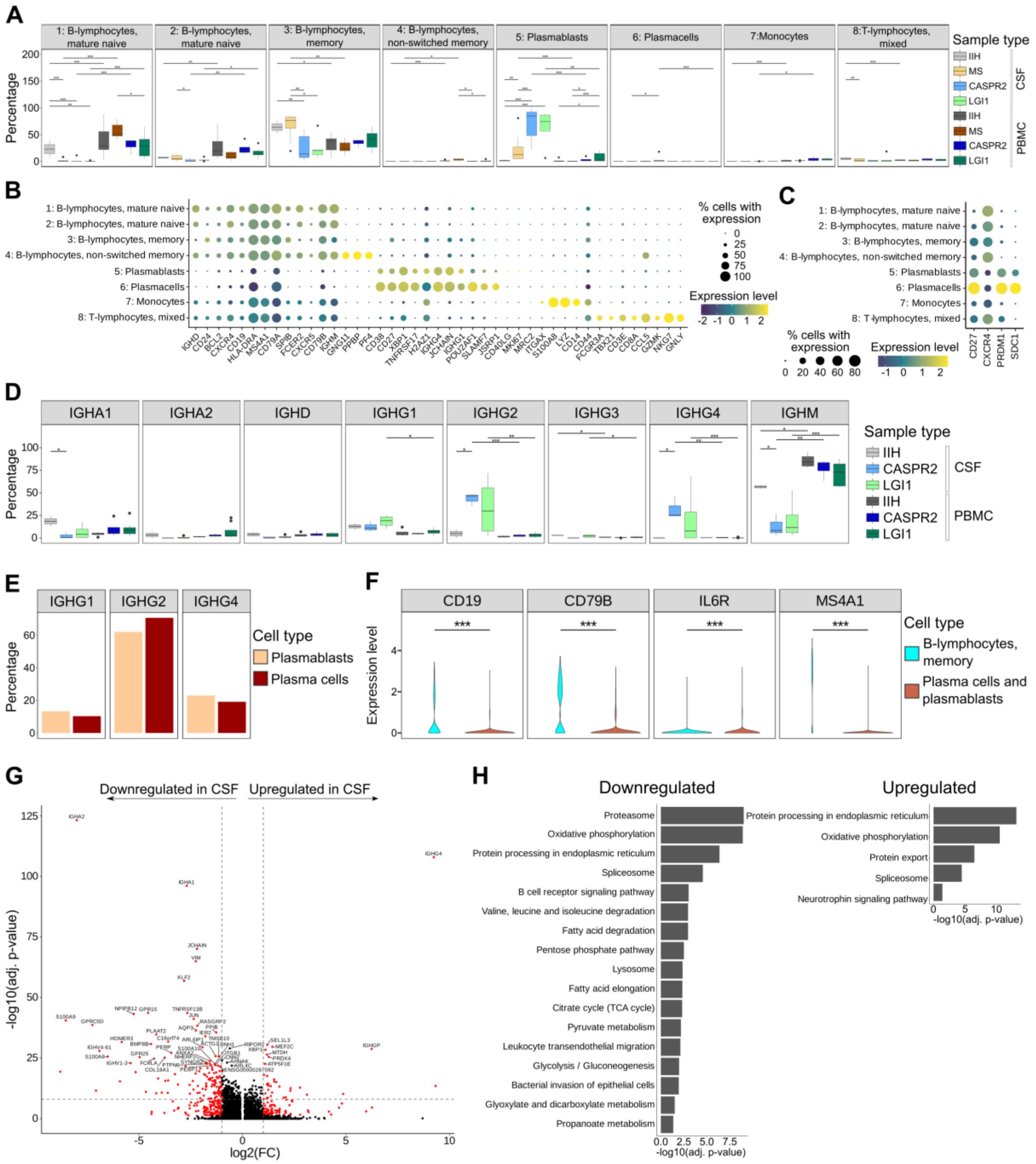

Fig. S3 ScRNAseq-based characterization of B-lineage cells including antibody-secreting cells (ASCs) and their respective BCR in CSF of patients with LGI1-/CASPR2-AIE and controls (cohort #1) (A) Boxplot comparing the cell type distribution for all sample groups in the B cell lineages. P-values were determined with the tool Propeller for all comparisons within each tissue and for each sample type between tissues. (B) Gene expression level of marker genes for the B cell subtypes. (C) Gene expression profile of lowly expressed B cell marker genes. Since genes expressed at low levels in very few cells were excluded for analysis, raw counts of these transcripts were investigated. (D) Comparison of the immunoglobulin subtypes. P-values were calculated with the tool propeller for all comparisons within each tissue and for each sample type between CSF cells and PBMCs. (E) Comparison of selected immunoglobulin subtypes between plasma cells and plasmablasts in

CSF from one LGI1 patient. In the other patients, the number of plasma cells was too low. (F) Gene expression level of selected genes relevant for therapeutic approaches in plasma cells / plasmablasts in comparison to memory B lymphocytes in CSF from AIE patients. Statistical significance was determined with the R package MAST. All adjusted p-values were lower than 10^{-5}. (G) Differentially expressed genes between CSF and PBMCs for plasmablasts and plasma cells. All genes with a fold change larger 2 and a p-value smaller 10^{-8} are marked in red. The 50 genes with the lowest p-value are labeled. (H) Gene set enrichment analyses based on downregulated (left plot) and upregulated (right plot) genes for the comparison between CSF and PBMCs in plasma cells/plasmablasts. Pathways, which were obviously disease-associated, are not shown to remove potential artifacts.

Figure S4:

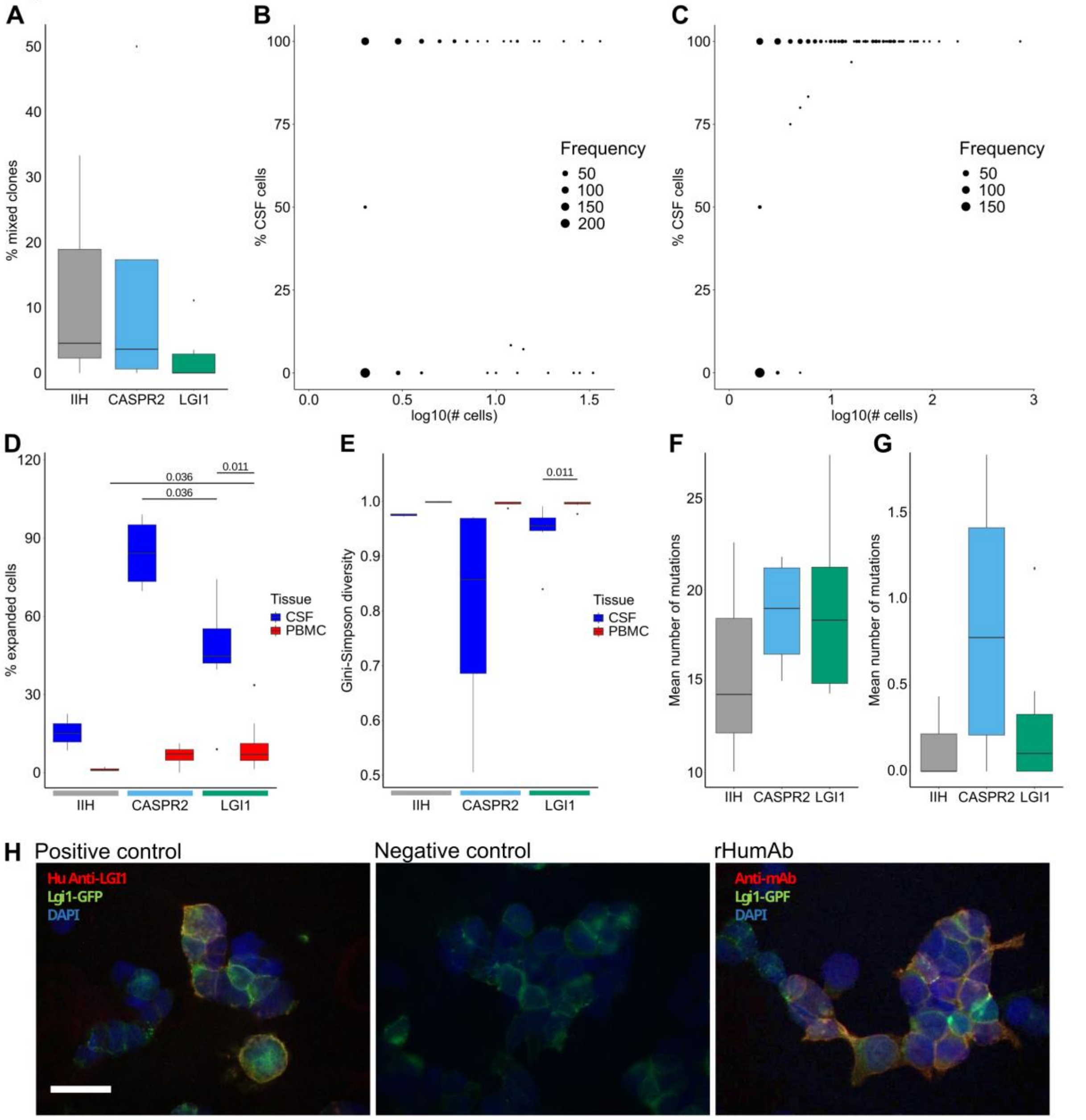

Fig. S4 Analysis of B cell receptors of ASCs in CSF of patients with LGI1-/CASPR2-AIE and verification of autoantigen-specificity (cohort #1)

(A) Percentage of “mixed” clones consisting of PBMC and CSF derived B cells. (B+C) The figures illustrate the proportion of CSF-to-PBMC derived members in each B cell clone in relation to the clone size for (B) LGI1 and (C) CASPR2 patients. (D) Percentage of clonally expanded B cells in the network. Statistical significance was determined with a Mann-Whitney-U test followed by Benjamini-Hochberg correction for all comparisons within each tissue and for each sample type between tissues. (E) Gini-Simpson index of the BCRs. (F) The average number of mutations on the heavy chain in the VDJ region in comparison to the closest common ancestor was determined per clone and the average number per sample split by sample type visualized in the figure. (G) Average number of intraclonal mutations per sample separated by sample type. (H) Verification of autoantigen specificity of rhumabs derived from expanded CSF plasma cell clones. Indirect immunofluorescence on HEK293T cells transfected with human full-length LGI1 c-terminally fused to a CASPR2 transmembrane

domain and intracellular GFP. Secondary staining using anti-Human IgG Alexa 569. Positive and negative control serum at 1:40 dilutions. Human IgG red, LGI1-transfected cells green, nuclear DAPI counterstaining blue. Scale bar 40µm.

Figure S5:

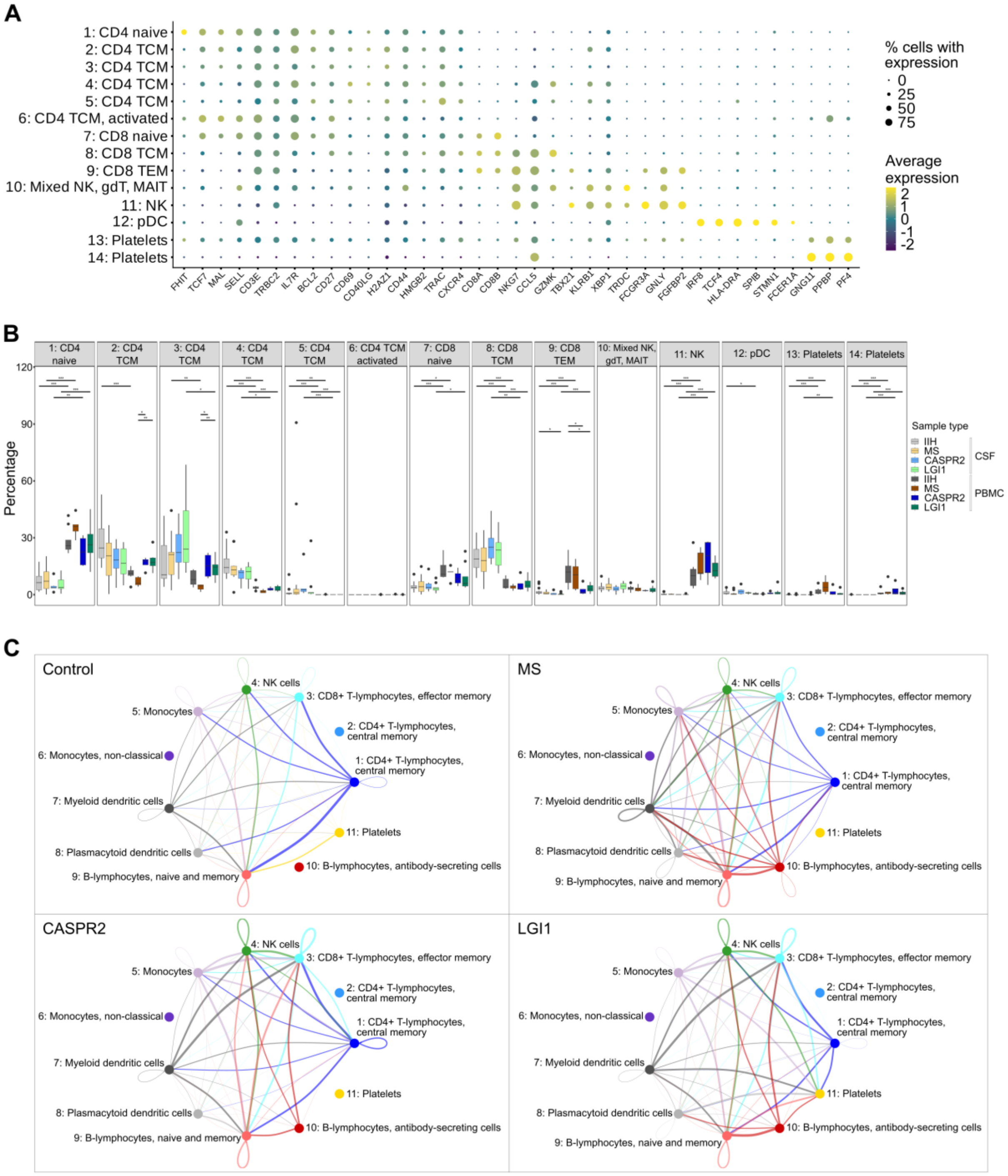

Fig. S5 Classification and cluster-based annotation of T lymphocytes in CSF and PBMCs of patients with LGI1-/CASPR2-AIE and controls (cohort #1)

(A) Gene expression level of marker genes for T cell lineages. (B) Distribution of T cell subtypes across all sample types and tissues. P-values were calculated with the tool propeller for all comparisons within each tissue and for each sample type between CSF cells and PBMCs. (C) Interactions between cell types in CSF.

Figure S6:

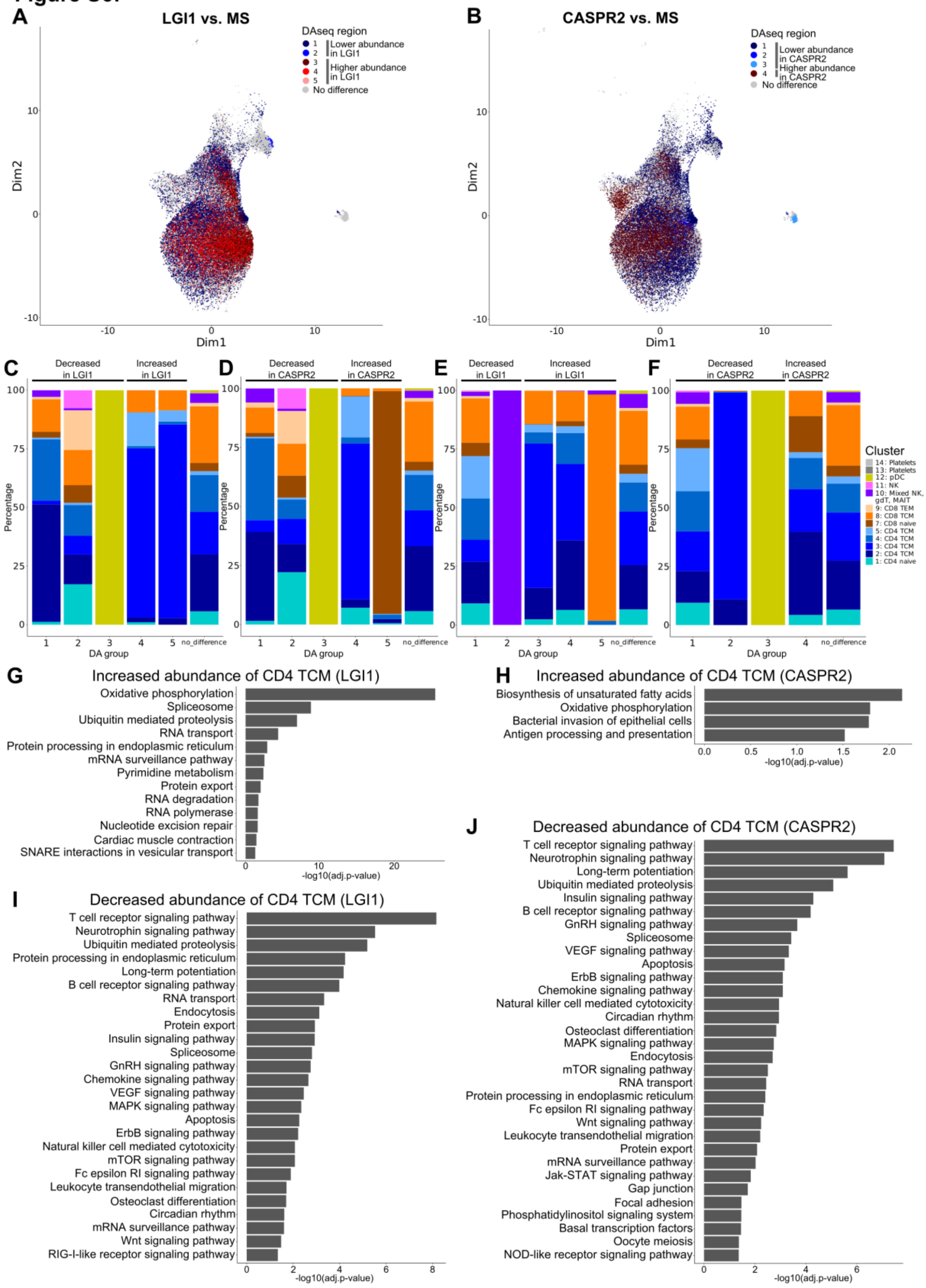
A
LGI1 vs. MS
DAseq region
1
2
Lower abundance in LGI1
3
4
5
Higher abundance in LGI1
No difference
Dim2
Dim1
B
CASPR2 vs. MS
DAseq region
1
2
Lower abundance in CASPR2
3
4
Higher abundance in CASPR2
No difference
C
Decreased in LGI1
Increased in LGI1
Percentage
DA group
no_difference
D
Decreased in CASPR2
Increased in CASPR2
E
Decreased in LGI1
Increased in LGI1
F
Decreased in CASPR2
Increased in CASPR2
Cluster
14: Platelets
13: Platelets
12: pDC
11: NK
10: Mixed NK, gdT, MAIT
9: CD8 TEM
8: CD8 TCM
7: CD8 naive
5: CD4 TCM
4: CD4 TCM
3: CD4 TCM
2: CD4 TCM
1: CD4 naive
G
Increased abundance of CD4 TCM (LGI1)
Oxidative phosphorylation
Spliceosome
Ubiquitin mediated proteolysis
RNA transport
Protein processing in endoplasmic reticulum
mRNA surveillance pathway
Pyrimidine metabolism
Protein export
RNA degradation
RNA polymerase
Nucleotide excision repair
Cardiac muscle contraction
SNARE interactions in vesicular transport
-log10(adj.p-value)
H
Increased abundance of CD4 TCM (CASPR2)
Biosynthesis of unsaturated fatty acids
Oxidative phosphorylation
Bacterial invasion of epithelial cells
Antigen processing and presentation
-log10(adj.p-value)
I
Decreased abundance of CD4 TCM (LGI1)
T cell receptor signaling pathway
Neurotrophin signaling pathway
Ubiquitin mediated proteolysis
Protein processing in endoplasmic reticulum
Long-term potentiation
B cell receptor signaling pathway
RNA transport
Endocytosis
Protein export
Insulin signaling pathway
Spliceosome
GnRH signaling pathway
Chemokine signaling pathway
VEGF signaling pathway
MAPK signaling pathway
Apoptosis
ErbB signaling pathway
Natural killer cell mediated cytotoxicity
mTOR signaling pathway
Fc epsilon RI signaling pathway
Leukocyte transendothelial migration
Osteoclast differentiation
Circadian rhythm
mRNA surveillance pathway
Wnt signaling pathway
RIG-I-like receptor signaling pathway
-log10(adj.p-value)
J
Decreased abundance of CD4 TCM (CASPR2)
T cell receptor signaling pathway
Neurotrophin signaling pathway
Long-term potentiation
Ubiquitin mediated proteolysis
Insulin signaling pathway
B cell receptor signaling pathway
GnRH signaling pathway
Spliceosome
VEGF signaling pathway
Apoptosis
ErbB signaling pathway
Chemokine signaling pathway
Natural killer cell mediated cytotoxicity
Circadian rhythm
Osteoclast differentiation
MAPK signaling pathway
Endocytosis
mTOR signaling pathway
RNA transport
Protein processing in endoplasmic reticulum
Fc epsilon RI signaling pathway
Wnt signaling pathway
Leukocyte transendothelial migration
Protein export
mRNA surveillance pathway
Jak-STAT signaling pathway
Gap junction
Focal adhesion
Phosphatidylinositol signaling system
Basal transcription factors
Oocyte meiosis
NOD-like receptor signaling pathway
-log10(adj.p-value)

Fig. S6 Cluster-free differential abundance analysis of T lymphocytes in CSF of patients with LGI1-/CASPR2-AIE and controls (cohort #1)

(A-B) DAseq regions in the comparison between (A) LGI1 and MS and (B) CASPR2 and MS in CSF T cells. (C-F) Cell type cluster composition of each DAseq group in the comparisons (C) LGI1 vs. IIH, (D), CASPR2 vs. IIH, (E) LGI1 vs. MS, (F) CASPR2 vs. MS. Cluster 6 does not include CSF cells and is therefore not shown. (G-J) Pathways enriched for genes with higher expression level in CD4-TCM DAseq regions with increased (G,H) or decreased (I,J) abundance in LGI1- (G,I) or CASPR2-AIE (H,J) relative to IIH controls.

Figure S7:

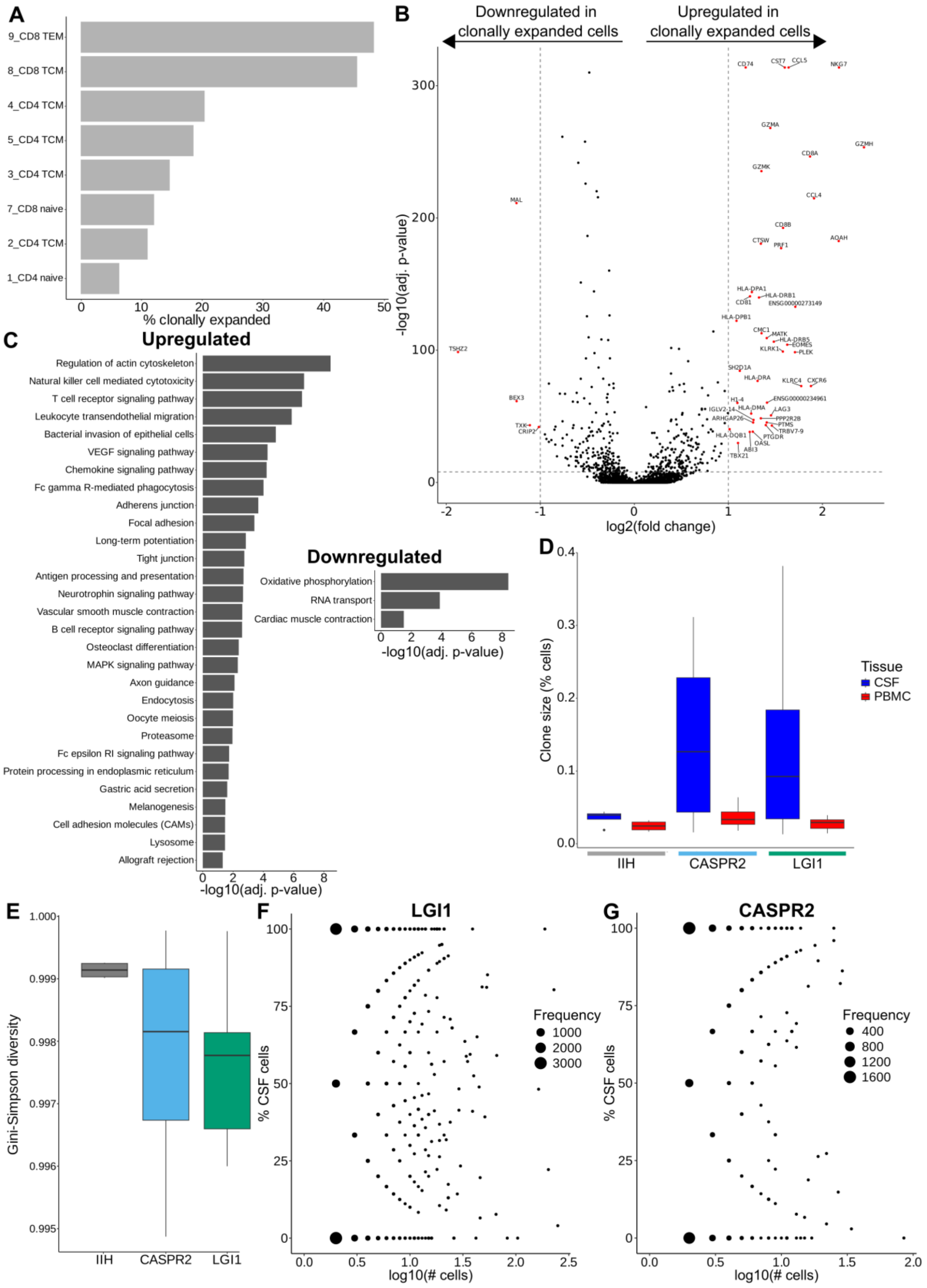
A
9_CD8 TEM
8_CD8 TCM
4_CD4 TCM
5_CD4 TCM
3_CD4 TCM
7_CD8 naive
2_CD4 TCM
1_CD4 naive
0 10 20 30 40 50
% clonally expanded
B
Downregulated in clonally expanded cells
Upregulated in clonally expanded cells
-log10(adj. p-value)
0 100 200 300
-2 -1 0 1 2
log2(fold change)
C
Upregulated
Regulation of actin cytoskeleton
Natural killer cell mediated cytotoxicity
T cell receptor signaling pathway
Leukocyte transendothelial migration
Bacterial invasion of epithelial cells
VEGF signaling pathway
Chemokine signaling pathway
Fc gamma R-mediated phagocytosis
Adherens junction
Focal adhesion
Long-term potentiation
Tight junction
Antigen processing and presentation
Neurotrophin signaling pathway
Vascular smooth muscle contraction
B cell receptor signaling pathway
Osteoclast differentiation
MAPK signaling pathway
Axon guidance
Endocytosis
Oocyte meiosis
Proteasome
Fc epsilon RI signaling pathway
Protein processing in endoplasmic reticulum
Gastric acid secretion
Melanogenesis
Cell adhesion molecules (CAMs)
Lysosome
Allograft rejection
0 2 4 6 8
-log10(adj. p-value)
Downregulated
Oxidative phosphorylation
RNA transport
Cardiac muscle contraction
0 2 4 6 8
-log10(adj. p-value)
D
Clone size (% cells)
0.0 0.1 0.2 0.3 0.4
Tissue
CSF
PBMC
IIH
CASPR2
LGI1
E
Gini-Simpson diversity
0.995 0.996 0.997 0.998 0.999 1.000
IIH
CASPR2
LGI1
F
LGI1
% CSF cells
0 25 50 75 100
0.0 0.5 1.0 1.5 2.0 2.5
log10(# cells)
Frequency
1000
2000
3000
G
CASPR2
% CSF cells
0 25 50 75 100
0.0 0.5 1.0 1.5 2.0
log10(# cells)
Frequency
400
800
1200
1600

Fig. S7 Description of clonally expanded T lymphocytes in CSF and shared clones between CSF/PBMCs of patients with LGI1-/CASPR2-AIE and controls (cohort #1)

(A) Percentage of clonally expanded cells per cell type. (B) Differentially expressed genes between clonally expanded and non-expanded T cells in CSF from AIE patients. (C) Pathways enriched for upregulated (left) and downregulated (right) genes in the comparison between clonally expanded and unexpanded T cells in CSF from AIE patients. (D) Comparison of the average clone sizes in each sample type. The clone size was determined by the percentage of cells in each clone in relation to all T cells with TCR information in the sample. (E) Gini Simpson diversity for T cells. (F-G) Proportion of CSF-to-PBMC derived members in each clone in relation to the clone size for (F) LGI1 and (G) CASPR2.

Figure S8:

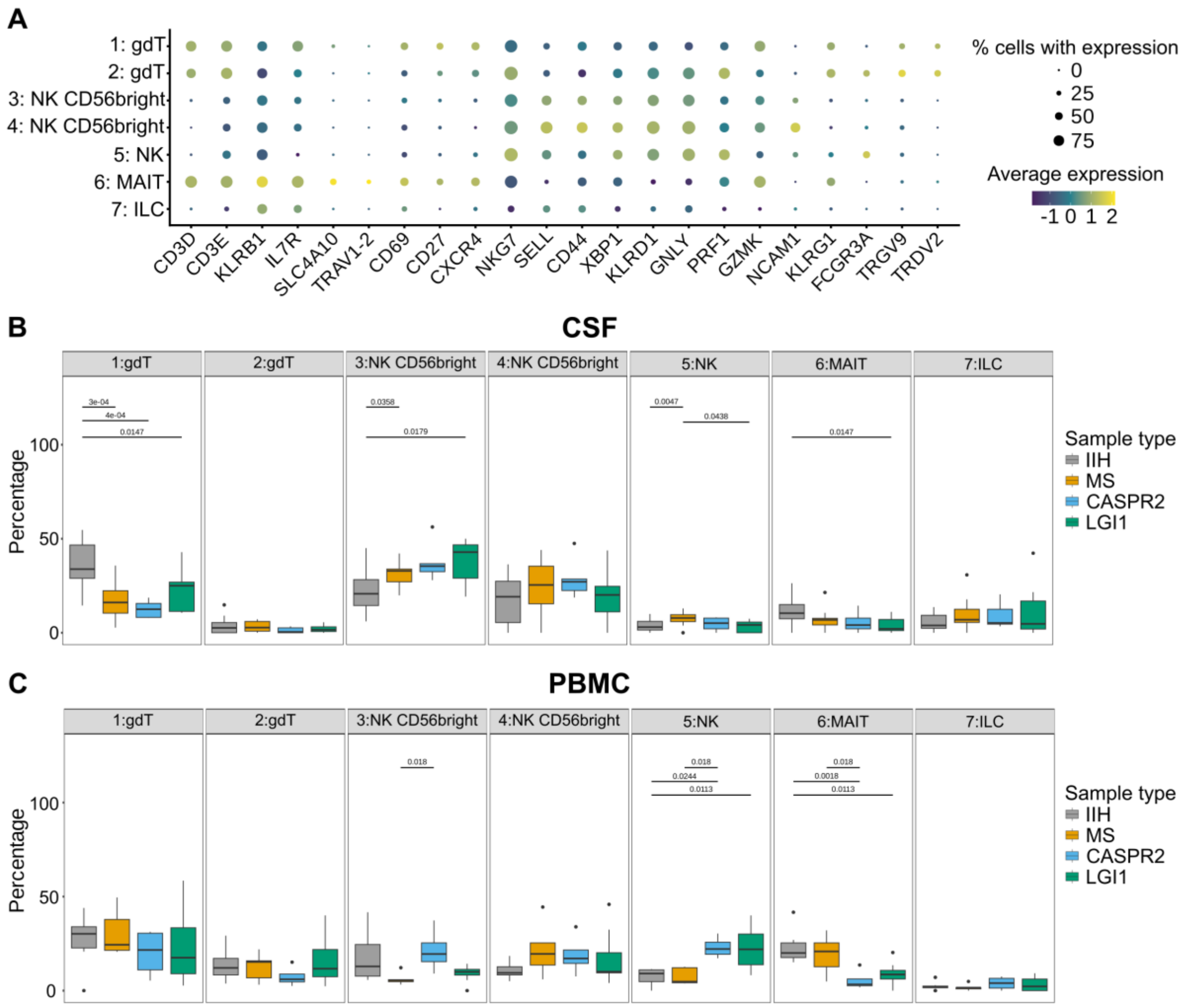

Fig. S8 Subcluster-based identification and annotation of invariant T cells and non-conventional lymphocytes in CSF and PBMCs of patients with LGI1-/CASPR2-AIE and controls (cohort #1)

(A) Expression level of marker genes for cell types in the subclustering of cells from T cell cluster "Mixed NK, gdT, MAIT". (B-C) Comparison of cell type distribution in the T cell subcluster "Mixed NK, gdT, MAIT" for (B) CSF and (C) PBMC.

Figure S9:

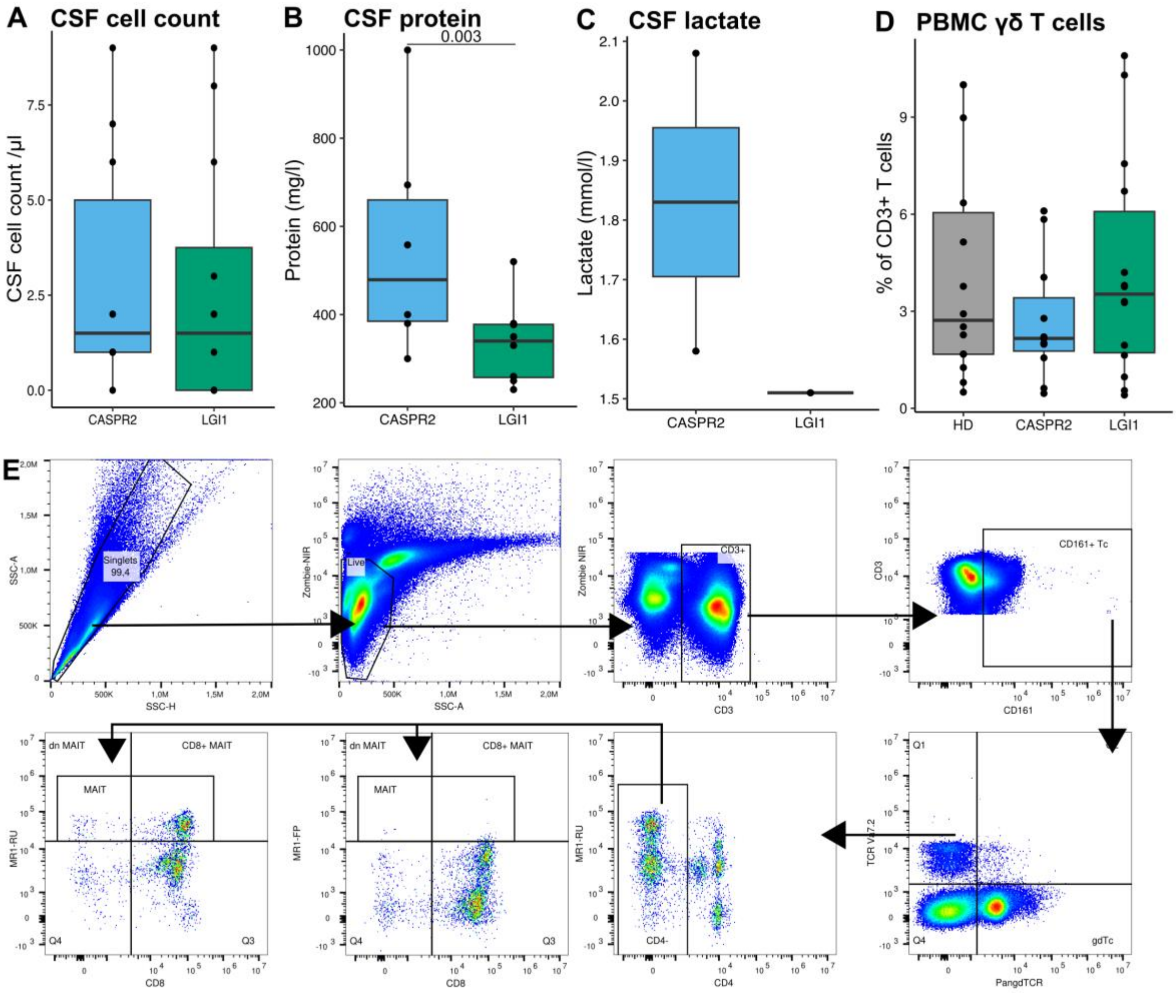

Fig. S9 Flow cytometry gating strategy, basic CSF features, and flow cytometry based cell frequencies of invariant T cells in PBMCs of patients with LGI1-/CASPR2-AIE and controls (cohort #3)

(A-C) Basic CSF parameters of cohort #3. (D) Proportion of gamma delta T cells quantified as percentages of all CD3+ T cells in CSF cells in the flow cytometry data. Statistical significance for (A-D) was determined by Kruskal-Wallis with post hoc Dunn’s test and Benjamini-Hochberg adjusted. (E) Representative flow cytometry gating is shown in pseudocolor plots. CSF cells were gated on Forward scatter and side scatter followed by marker genes as shown.

Figure S10:

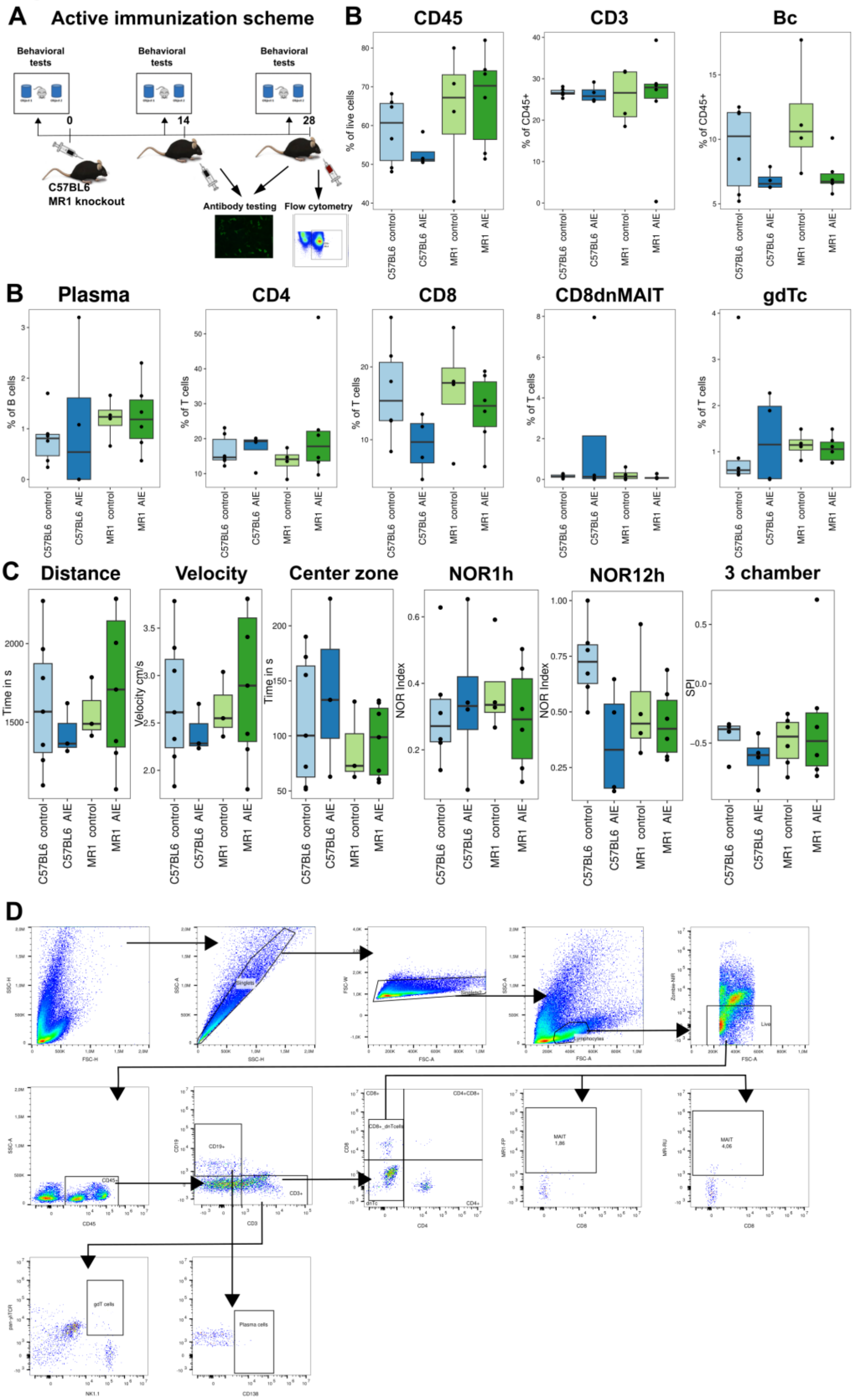
A
Active immunization scheme
Behavioral tests
Behavioral tests
Behavioral tests
0
14
28
C57BL6
MR1 knockout
Antibody testing
Flow cytometry
B
CD45
CD3
Bc
% of live cells
% of CD45+
C57BL6 control
C57BL6 AIE
MR1 control
MR1 AIE
B
Plasma
CD4
CD8
CD8dnMAIT
gdTc
% of B cells
% of T cells
C
Distance
Velocity
Center zone
NOR1h
NOR12h
3 chamber
Time in s
Velocity cm/s
NOR Index
SPI
D

Fig. S10 Immunization strategy, behavioral and whole-brain flow cytometry in the murine LGI1/CASPR2-immunization model.

(A) Representative scheme of murine immunization model (Suppl. methods) of 12 MR1 deficient and 10 C57BL/6 mice. Serum and brain tissue samples were analyzed and mice underwent three behavioral tests across three timepoints. (B) Flow cytometric analysis of the CNS of immunized (n=10) and control mice (n=10). (C) Analysis of the open field test (distance, velocity, time in center zone), Novel object recognition test and three chamber test shown in barplots. (D) Representative flow cytometry gating is shown in pseudocolor plots. CNS leukocytes were gated on forward scatter and side scatter followed by marker genes as shown. The statistical significance was calculated with the Kruskal-Wallis test with post-hoc Dunn's test. The p values were adjusted with the Benjamini-Hochberg's method.
