# Supplementary material for: Activated αβ T and reduced mucosa-associated invariant T cells in LGI1- and CASPR2-encephalitis
Source: Brain. 2025 Mar 17;148(9):3170–83. doi: 10.1093/brain/awaf096 (PMC12404778; doi:10.1093/brain/awaf096)
Supplement: awaf096_Supplementary_Data [file awaf096_supplementary_data.zip › brain-2024-00707-File008.pdf]

EMC AIE study group

Juna M de Vries, MD PhD[1]	j.m.devries@erasmusmc.nl	ORCID ID: 0000-0002-6380-1871
Mariska M.P. Nagtzaam, BSc[1]	m.nagtzaam@erasmusmc.nl	ORCID ID: 0009-0007-9427-0835
Suzanne C. Franken, MSc[1]	s.c.franken@erasmusmc.nl	ORCID ID: 0009-0006-7582-5295
Yvette S. Crijnen, MD[1]	y.crijnen@erasmusmc.nl	ORCID ID: 0000-0003-0279-7140
Juliette Brenner, MD[1]	j.brenner@erasmusmc.nl	ORCID ID: 0000-0001-5523-4398
Robin W. van Steenhoven, MD[1]	r.vansteenhoven@erasmusmc.nl	ORCID ID: 0009-0003-6842-095X
Jeroen Kerstens, MD[1]	j.kerstens@erasmusmc.nl	ORCID ID: 0000-0002-3185-6974
Marienke A.A.M. de Bruijn, MD PhD[1]	m.debruijn@erasmusmc.nl	
Anna E.M. Bastiaansen, MD PhD[1]	a.bastiaansen@erasmusmc.nl	ORCID ID: 0000-0002-8492-5638
Remco M. Hoogenboezem[2]MSC	r.hoogenboezem@erasmusmc.nl	ORCID ID: 0000-0002-1719-6455
Sharon Veenbergen, PhD[3]	s.veenbergen@erasmusmc.nl	
Peter A.E. Sillevis Smitt, MD PhD[1]	p.sillevissmitt@erasmusmc.nl	ORCID ID: 0000-0001-8044-6798

[1]Department of Neurology, Erasmus University Medical Center, Rotterdam, The Netherlands
[2]Department of Hematology, Erasmus University Medical Center, Rotterdam, The Netherlands
[3]Department of Immunology, Erasmus University Medical Center, Rotterdam, The Netherlands
