# Supplementary material for: Activated αβ T and reduced mucosa-associated invariant T cells in LGI1- and CASPR2-encephalitis
Source: Brain. 2025 Mar 17;148(9):3170–83. doi: 10.1093/brain/awaf096 (PMC12404778; doi:10.1093/brain/awaf096)
Supplement: awaf096_Supplementary_Data [file awaf096_supplementary_data.zip › brain-2024-00707-File015.pdf]

Supplemental material

Activated αβ T- and reduced mucosa-associated invariant T-cells in LGI1- and CASPR2-encephalitis

Authors:

Daniela Esser[1,*], Louisa Müller-Miny[2,*], Michael Heming[2,*], Manuela Paunovic[3], Martijn van Duijn[3] (0000-0002-6654-994X), Ligia Abrante Cabrera[1], Katharina M Mair[6], Christine Strippel[2], Saskia Räuber[2,5], Justina Dargvainiene[1], Stjepana Kovac[2] (0000-0002-2500-6893) Catharina C. Gross[2] (0000-0002-4872-9189), Nina Fransen[8], Robin van Steenhoven[3], Péter Körtvélyessy[9](0000-0003-1262-6169), Werner Stenzel[10], Hans Arnholdt[11], Romana Höftberger[6], Eric Bindels[4], Heinz Wiendl[12], Sven G. Meuth[2,5], Jan Bauer[6], Nico Melzer[2,5] (0000-0002-2420-701X), Maarten J. Titulaer[3] (0000-0002-1033-3840), Frank Leypoldt[1,7,§] (0000-0002-8972-515X) and Gerd Meyer zu Hörste[2,§] (0000-0002-4341-4719) in cooperation with the EMC-AIE Study group

Supplemental methods

Retrospective sampling in cohort 2

We retrospectively screened all patients who were admitted to the University Hospital Münster between 2011 and 2020 and received a diagnostic lumbar puncture, including flow cytometry, for the ICD-10 diagnoses G04.*, G13.1, G93.2. Flow cytometry raw data from all treatment-naive patients was analyzed (Suppl. Fig. 2).

All CSF cell samples collected during regular working hours at the center in Münster were routinely and promptly analyzed by flow cytometry using a Navios flow cytometer (Beckman Coulter) and an antibody panel described previously [1]. Briefly, blood cells were lysed using VersaLyse buffer and blood and CSF cells were stained using the following anti-human antibodies (Biolegend; clone names indicated): CD3 (UCHT1); CD4 (13B8.2); CD8 (B9.11); CD14 (RMO52); CD16 (3G8); CD19 (J3-119); CD25 (B1.49.9); CD27 (1A4CD27); CD45 (J.33); CD45RA (ALB11); CD56 (N901, NCAM16.2); CD127 (R34.34); CD138 (B-A38); and HLA-DR (Immu-357). The gating scheme is depicted in (Suppl. Fig. 2A). Cell population size was defined as the number of gated cell events relative to the events of the corresponding parent gate.

Retrospective sampling in cohort 3

For blood flow cytometry, we retrospectively screened available cryo-preserved PBMCs of patients with confirmed LGI1-/CASPR2-AIE at the center in Münster. Peripheral blood was cryopreserved, as described previously [2]. Frozen PBMC were thawed, divided into separate samples and stained with distinct sets of fluorochrome-conjugated antibodies over 30 minutes with anti-human antibodies as indicated. T cell panel consisted of pan-γδTCR (B1), CD3 (OKT3), CD161 (HP3G10), TCRVα7.2 (3C10), CD19(SJ25C1), CD4 (SK2), CD8a (HIT8a), 7-AAD, Tetramer MR1-RU, and MR1-FP[3] as control staining. The MR1 tetramer technology was developed jointly by Dr. James McCluskey, Dr. Jamie Rossjohn, and Dr. David Fairlie, and the material was produced by the NIH Tetramer Core Facility as permitted to be distributed by the University of Melbourne. Flow-cytometric data were analyzed using FlowJo V10. Immune cell subsets were defined according to a prespecified gating hierarchy (Suppl. Fig. 9).

Exclusion criteria in cohort1

Exclusion criteria for all patients in cohort #1 were defined as: (1) questionable diagnosis of AIE by clinical signs or magnetic resonance imaging (MRI) findings, and (2) ongoing or previous immunomodulatory treatment. IIH patients were included, if they gave informed consent. Exclusion criteria for all patients were: (1) immunologically relevant comorbidities (e.g. rheumatologic diseases), (2) severe concomitant infectious diseases (e.g. HIV, meningitis, and encephalitis), (3) pregnancy or breastfeeding, (4) younger than 18 years, (5) mental illness impairing the ability to give informed consent, and (6) artificial blood contamination during the LP resulting in >200 RBCs/µl.

CSF for scRNA-seq was collected as described [4]. Briefly, during LPs performed for clinical reasons, up to 10 ml of CSF and 3ml of blood were collected in addition to diagnostic material. CSF was transported at 4°C and processed within an hour to ensure optimal sample quality. CSF cells, total protein, and intrathecal immunoglobulin concentrations were assessed according to validated standard diagnostic procedures in central labs of each center. The concentration of protein and immunoglobulins in serum and CSF were compared and a Reiber scheme was created to evaluate the integrity of the blood-CSF-barrier (BCBD), quantified by the ratio between CSF albumin and serum albumin. Oligoclonal bands (OCB) were detected by isoelectric focusing and silver nitrate staining. Samples were pseudonymized at collection. CSF was centrifuged at 300×g for 10 min. The supernatant was removed. CSF cells were resuspended in 5ml of X-Vivo15 media (Lonza). A total of 5 µl of the single-cell suspension were manually counted in a counting chamber. The remainder of the CSF cells and a maximum of 20,000 CSF were used as input for scRNA-seq. If total available CSF cell numbers were <17,000 cells, all available cells were processed.

Antibody Titration

Antibody positivity was determined by commercial (Euroimmun, Lübeck) cell-based- and specialized rat brain tissue based assays as previously described. [5-8] Titrations were performed with cell-based assays (Euroimmun CBA) and/or rat brain tissue based assays) as previously described (Suppl. Table 1).

Cell abundance analysis

Differentially abundant cell type clusters were determined with the function propeller[9] included in the R package speckle v1.4.0. Clusters with less than 30 cells in both conditions combined were removed. For T cells, sub-populations with differential abundance between conditions were identified with a cluster-free approach using the tool DA-seq [10] v1.0.0 based on the Harmony normalized data with a threshold of 0.8.

Differential gene expression and enrichment analysis

Differentially expressed genes were determined using the FindMarkers function with MAST v1.30.0 [11]. Thereby, only genes with a minimum fraction of 0.1 in either of the two groups were considered. In the volcano plots, p-values equal to zero were adapted to the lowest p-value>0 in the data set to enable the calculation of logarithmic values. The gene set enrichment analyses were performed with the online tool InnateDB [12] based on entries from the KEGG [13] database. For visualization, obvious disease-associated pathways were excluded to remove artifacts. Ligand-receptor interactions between B and T cells were calculated with the R tool CellChat v2.1.2 based on the subset 'Secreted Signaling' of the human CellChatDB database and the 'weight' option [14].

BCR and TCR bioinformatic analysis

The preprocessing of the VDJ sequencing data was performed with 10x Genomics Cell Ranger software v8.0.1 with the VDJ reference GRCh38 v3.1.0. Only receptor sequences classified as cell, productive, and full-length were considered for further analyses. The BCR networks were based on the IGH chain. Cells were merged to one clone, if they harbored the same variable region. Clones were connected, if they had, based on the Cellranger output, the same variable (V), joining (J), and constant (C) genes, the same CDR3 length and the CDR3 sequences differed not more than two nucleotides. In the TCR networks, cells formed one clone, if they had the same V, J, and C genes as well as the same CDR3 region on the TRB chain. Based on the network clones, the Gini-Simpson diversity was determined with the diversity function of the R microbiome package v1.23.1. The clone sizes reported by Cellranger were visualized in the UMAP plots and used to investigate the clonal expansion level per cell type. The immunoglobulin subtypes of the B cells and the number of mutations in relation to a common ancestor were determined with IgBLAST[15] from NCBI based on the IMGT[16] references and the NCBI database.

Cloning antibodies and verification of antigen-specificity

Corresponding full length consensus sequences of variable heavy (VH) and light chains (VL) including signal peptide sequences of the top 5-9 most abundant, clonally expanded B cells in the CSF sample of five randomly selected patients (3 LGI1, 2 CASPR2-AIE) were determined from 5' VDJ 10x single cell information. Cloning strategy, vectors, and expression/purification strategies were kindly provided by M. Peipp, Kiel and done as previously described [17]. All sequences were synthesized by GeneArt (ThermoFisher) and cloned into pcDNA3.1(+) expression vectors. Human Embryonic Kidney (HEK-293) cells were transfected and supernatant incubated with CaptureSelect™ IgG-CH1 Affinity Matrix (Thermo Fisher 1943200250). Antibodies were eluted by acidic elution and neutralized. Purified recombinant human antibodies (rHumAbs) were desalted (Zeba™ Spin Desalting Columns, Thermo Fisher 89891) and concentrated using a Christ Rotational Vacuum Concentrator System. Antibody concentration was determined with Qbit protein assay (Invitrogen Q33211). Antigen-specificity was confirmed using cell-based assays (CBA) as previously reported [5,6] with slight modifications. HEK-293 cells were transfected with a plasmid encoding full-length human LGI1 c-terminally fused to a transmembrane CASPR2 domain and intracellular GFP, or full-length human CASPR2 with C-terminal GFP tag (both kindly provided by S. Irani, Oxford). Secondary staining was done using a goat anti-Human Alexa Fluor (AF) 594 antibody (1:1000, Invitrogen). Cells were mounted in Vectashield HardSet Mounting Medium with DAPI (4′,6-diamidino-2-phenylindole, Vector H-1500), and results were evaluated using a fluorescence microscope. Human serum (1:40) from autoimmune encephalitis patients or healthy subjects was used as positive and negative controls, respectively.

Human CNS multiplex immunofluorescence labeling

Histological paraffin sections of brain and meningeal tissue from autopsied patients with the diagnosis CASPR2-AIE (n=3) and LGI1-AIE (n=3) were included. Sections were localized in the uncal and hippocampal region. Immunofluorescence labeling of sections was performed using markers for CD3 (Neomarkers, #RM9107-S), CD8 (Dako M7103, CD4 (Cell Signailing, #48274), TCR δ(SantaCruz, #sc-100289) and CD161 (Abcam, #ab302564). The staining procedure was executed in accordance with the Akoya Fluorescent Multiplex kit protocol[18,19]. In short, antigen retrieval was achieved by placing samples in EDTA pH9 for 60 minutes in a

household food steamer (Braun), followed by a 10-minute incubation with Opal Antibody Dilutent/Block solution (Akoya Bioscience, Marlborough, USA). The first primary antibody was then applied overnight at 4°C. Subsequently, sections were rinsed several times in Tris-buffer saline with Tween 20 (TBS-T) and the secondary antibody was applied. Hence either horseradish peroxidase (HRP) conjugated donkey α-mouse (Jackson, #715-225-151) or HRP conjugated donkey α-rabbit (Jackson, #711-035-152) was used. Afterwards, one of the fluorophores was introduced (Opal 570, Opal 690, Opal 480, Opal 620). Before proceeding with the next primary antibody, the samples were fixed with 4% paraformaldehyde for 10 minutes at RT followed by another antigen retrieval step with AR6 for 30 minutes. Regarding Opal 780, the staining procedure differed: after incubation with the secondary antibody, Opal TSA-DIG (Akoya Bioscience, Marlborough, USA) was introduced for 10 minutes. Next, the sections were transferred into AR6 and heated in a household food steamer for 20 minutes. The fluorophore signal was generated by incubating the sections with Opal 780 at RT for 60 minutes and 4′,6-diamidino-2-phenylindole (DAPI) was applied for counterstaining.

To quantify, cells were scanned with the Vectra Polaris Automated Quantitative Pathology Imaging system from Perkin Elmer and quantified with Qupath software. To this end, cells were detected by nuclear staining (DAPI).

Mouse immunisation

All animal experiments were approved by the local authorities (Landesamt für Natur, Umwelt und Verbraucherschutz Nordrhein-Westfalen; Approval ID: 84-02.04.2022.A336). Every effort was made to minimize the number of animals used and to avoid stress and suffering of the animals by strictly following the ARRIVE guidelines. The mice were housed in groups, had a 12-hour light/dark cycle, and food and water were available ad libitum. Homozygous MR1 deficient (MR1AIE; LGI1 n=3, CASPR2 n=5) and C57BL/6 (C57BL6AIE; LGI1 n=2; CASPR2 n=5) mice (10-15 weeks old, male and female) were immunized twice with Recombinant Mouse CNTNAP2 (>95% purity) or LGI1 protein (>90% purity) (Cntnap2-3316M, LGI1-9069M, Creative Biomart) emulsified in Complete Freund's Adjuvant (CFA) and supplemented with Mycobacterium tuberculosis H37Ra (4 mg/mL). Mice were immunized subcutaneously on the back with 100 µg of the protein in the emulsion mixture. Mice in the control groups, including homozygous MR1 deficient (MR1control; n=5) and C57BL/6 (C57BL6control; n=5) mice, received an emulsion mixture of CFA and the same volume of phosphate-buffered saline (PBS). All mice were injected intraperitoneally with 250 ng of pertussis toxin (Sigma) on the day of immunization and 48 hours later. Serum from all mice was collected on the 28th day to determine antibody titers.

Murine evaluation

Mice were evaluated 3-1 days prior, at 12-14 days and at 25-28 days after immunization and underwent behavioral tests to evaluate locomotor activity, anxiety levels and memory. All tests were evaluated by an automated program (Noldus Ethovision[20]) with manual correction.

The Open Field test assessed locomotor activity and exploratory behavior of mice[21]. Animals were tested in the open field arena and traveled distance, velocity, and time spent in the center were measured.

The Novel object recognition test (NOR) [22,23] was used to assess cognitive and memory functions. The same arena was used as animals were already familiar with it. After 3 familiarization sessions over 3 days animals were able to explore the arena with 2 identical objects for 5 minutes. Two test phases were tested to evaluate short term (1 hour) and long term (24 hours) memory [24]. For the testing session one of the familiar objects was replaced by

a novel object. The time spent exploring the novel and old object was used to calculate the Novel object recognition index (NOR Index: (time novel/ (time novel+time old)).
The three-chamber test [25,26] was performed in a plastic apparatus (60x60cm) with 3 chambers delimited by removable dividers after habituation a day prior. Testing was performed in 2 phases. Phase 1 included a 10 minute acclimation phase with 2 empty cages in the outer chambers. In phase 2 an empty cage(non-social stimulus) and a cage with an unfamiliar mouse (of the same sex) (social stimulus) was placed in the outer chambers.Time spent in each chamber was recorded over 10 minutes. The social preference index was calculated (social stimulus-non social stimulus/(social stimulus+nonsocial stimulus).
Mouse Serum of day 28 was extracted from the facial vein, centrifuged for 10 minutes and analyzed for the presence of IgG autoantibodies against neural surface membrane antigens (NMDAR, AMPAR, GABA, LGI1, CASPR2, DPPX) using a cell-based assay according to manufacturer's instructions (EUROIMMUN, Lübeck, Germany) with a secondary Anti-mouse-IgG Antibody (Biolegend, Poly4053). Dilution steps included pure, 1:1, 1:10, 1:32, 1:100,1:1000 according to standard clinical testing. Positivity for fluorescence was determined blinded on a Zeiss Axioscope (AX10).
Mice were perfused intracardially. The brain was extracted, digested with collagenase D (2.5 mg/mL) and DNase I (0.05 mg/mL) (20 min, 37 °C) and leukocytes were purified using a 70/37% Percoll gradient. Cells were stained for flow cytometry with anti-mouse antibodies (Biolegend; clone names indicated): CD45(30-F11), CD3 (17A2), CD4 (GK1.5), gdTCR (GL3), NK1.1 (S17016D) and CD11b (M1/70). Live/dead staining was performed with Zombie NIR. Anti-Mouse MR1 Tetramer MR1-RU [3] and control MR1-FP stainings were performed. Tetramer gating was introduced through a negative control for each sample (Suppl. Fig. 10). The MR1 tetramer technology was developed jointly by Dr. James McCluskey, Dr. Jamie Rossjohn, and Dr. David Fairlie, and the material was produced by the NIH Tetramer Core Facility as permitted to be distributed by the University of Melbourne. Cells were analyzed using an Aria flow cytometer (Beckman Coulter). Data were analyzed with FlowJo v10

Flow cytometric and murine statistics

Significance was tested with a Kruskal Wallis and post hoc Dunn test to compare multiple groups and the Benjamini Hochberg method used for multiple testing corrections. If two groups were compared a Mann-Whitney U test was used. Overall Fishers test was used to show significance in antibody incidence and post hoc individual Fishers test were performed to assess subgroup significance. The computational analysis was carried out with R 4.1.1.

References

1. Heming M, Müller-Miny L, Rolfes L, et al. Supporting the differential diagnosis of connective tissue diseases with neurological involvement by blood and cerebrospinal fluid flow cytometry. *J Neuroinflammation*. 2023;20(1):46. doi:10.1186/s12974-023-02733-w
2. Gross CC, Schulte-Mecklenbeck A, Madireddy L, et al. Classification of neurological diseases using multi-dimensional CSF analysis. *Brain*. 2021;144(9):2625-2634. doi:10.1093/brain/awab147
3. Corbett AJ, Eckle SBG, Birkinshaw RW, et al. T-cell activation by transitory neo-antigens derived from distinct microbial pathways. *Nature*. 2014;509(7500):361-365. doi:10.1038/nature13160
4. Schafflick D, Xu CA, Hartlehnert M, et al. Integrated single cell analysis of blood and cerebrospinal fluid leukocytes in multiple sclerosis. *Nature Communications*. 2020;11(1):247. doi:10.1038/s41467-019-14118-w

5. Lai M, Huijbers MG, Lancaster E, et al. Investigation of LGI1 as the antigen in limbic encephalitis previously attributed to potassium channels: a case series. *The Lancet Neurology*. 2010;9(8):776-785. doi:10.1016/S1474-4422(10)70137-X
6. Lancaster E, Huijbers MGM, Bar V, et al. Investigations of caspr2, an autoantigen of encephalitis and neuromyotonia. *Annals of Neurology*. 2011;69(2):303-311. doi:10.1002/ana.22297
7. Gresa-Arribas N, Titulaer MJ, Torrents A, et al. Antibody titres at diagnosis and during follow-up of anti-NMDA receptor encephalitis: a retrospective study. *Lancet Neurol*. 2014;13(2):167-177. doi:10.1016/S1474-4422(13)70282-5
8. Bastiaansen AEM, De Bruijn MAAM, Schuller SL, et al. Anti-NMDAR Encephalitis in the Netherlands, Focusing on Late-Onset Patients and Antibody Test Accuracy. *Neurol Neuroimmunol Neuroinflamm*. 2022;9(2):e1127. doi:10.1212/NXI.0000000000001127
9. Phipson B, Sim CB, Porrello ER, Hewitt AW, Powell J, Oshlack A. propeller: testing for differences in cell type proportions in single cell data. *Bioinformatics*. 2022;38(20):4720-4726. doi:10.1093/bioinformatics/btac582
10. Zhao J, Jaffe A, Li H, et al. Detection of differentially abundant cell subpopulations in scRNA-seq data. *Proc Natl Acad Sci USA*. 2021;118(22):e2100293118. doi:10.1073/pnas.2100293118
11. Finak G, McDavid A, Yajima M, et al. MAST: a flexible statistical framework for assessing transcriptional changes and characterizing heterogeneity in single-cell RNA sequencing data. *Genome Biol*. 2015;16(1):278. doi:10.1186/s13059-015-0844-5
12. Lynn DJ, Winsor GL, Chan C, et al. InnateDB: facilitating systems-level analyses of the mammalian innate immune response. *Molecular Systems Biology*. 2008;4(1):218. doi:10.1038/msb.2008.55
13. Kanehisa M, Furumichi M, Sato Y, Kawashima M, Ishiguro-Watanabe M. KEGG for taxonomy-based analysis of pathways and genomes. *Nucleic Acids Research*. 2023;51(D1):D587-D592. doi:10.1093/nar/gkac963
14. Jin S, Guerrero-Juarez CF, Zhang L, et al. Inference and analysis of cell-cell communication using CellChat. *Nat Commun*. 2021;12(1):1088. doi:10.1038/s41467-021-21246-9
15. Ye J, Ma N, Madden TL, Ostell JM. IgBLAST: an immunoglobulin variable domain sequence analysis tool. *Nucleic Acids Research*. 2013;41(W1):W34-W40. doi:10.1093/nar/gkt382
16. Lefranc MP, Giudicelli V, Ginestoux C, et al. IMGT, the international ImMunoGeneTics database. *Nucleic Acids Research*. 1999;27(1):209-212. doi:10.1093/nar/27.1.209
17. Kellner C, Derer S, Klausz K, et al. Fc Glyco- and Fc Protein-Engineering: Design of Antibody Variants with Improved ADCC and CDC Activity. In: Nevoltris D, Chames P, eds. *Antibody Engineering*. Vol 1827. Methods in Molecular Biology. Springer New York; 2018:381-397. doi:10.1007/978-1-4939-8648-4_20
18. Tröscher AR, Mair KM, Verdú De Juan L, et al. Temporal lobe epilepsy with GAD antibodies: neurons killed by T cells not by complement membrane attack complex. *Brain*. 2023;146(4):1436-1452. doi:10.1093/brain/awac404
19. Frieser D, Pignata A, Khajavi L, et al. Tissue-resident CD8 $^+$ T cells drive compartmentalized and chronic autoimmune damage against CNS neurons. *Sci Transl Med*. 2022;14(640):eabl6157. doi:10.1126/scitranslmed.abl6157
20. Noldus LPJJ, Spink AJ, Tegelenbosch RAJ. EthoVision: A versatile video tracking system for automation of behavioral experiments. *Behavior Research Methods, Instruments, & Computers*. 2001;33(3):398-414. doi:10.3758/BF03195394
21. Kraeuter AK, Guest PC, Sarnyai Z. The Open Field Test for Measuring Locomotor Activity and Anxiety-Like Behavior. In: Guest PC, ed. *Pre-Clinical Models*. Vol 1916. Methods in Molecular Biology. Springer New York; 2019:99-103. doi:10.1007/978-1-4939-8994-2_9
22. Leger M, Quiedeville A, Bouet V, et al. Object recognition test in mice. *Nat Protoc*. 2013;8(12):2531-2537. doi:10.1038/nprot.2013.155
23. Seibenhener ML, Wooten MC. Use of the Open Field Maze to Measure Locomotor and

Anxiety-like Behavior in Mice. *JoVE*. 2015;(96):52434. doi:10.3791/52434

24. Antunes M, Biala G. The novel object recognition memory: neurobiology, test procedure, and its modifications. *Cogn Process*. 2012;13(2):93-110. doi:10.1007/s10339-011-0430-z
25. Yang M, Silverman JL, Crawley JN. Automated Three-Chambered Social Approach Task for Mice. *CP Neuroscience*. 2011;56(1). doi:10.1002/0471142301.ns0826s56
26. Rein B, Ma K, Yan Z. A standardized social preference protocol for measuring social deficits in mouse models of autism. *Nat Protoc*. 2020;15(10):3464-3477. doi:10.1038/s41596-020-0382-9
